# Synthesis of a Small Library of Glycoderivative Putative Ligands of SGLT1 and Preliminary Biological Evaluation

**DOI:** 10.3390/molecules29215067

**Published:** 2024-10-26

**Authors:** Giuseppe D’Orazio, Barbara La Ferla

**Affiliations:** 1 Department of Chemistry, Università degli Studi di Milano, Via C. Golgi 19, 20133 Milan, Italy; 2 Department of Earth and Environmental Sciences DISAT, Università degli Studi di Milano-Bicocca, Piazza della Scienza 1, 20126 Milan, Italy

**Keywords:** sodium–glucose co-transporter 1, sugar absorption, chemotherapy-induced mucositis, inflammation, glycomimetics, glycoderivatives, *C*-glycosides

## Abstract

Sodium–glucose co-transporter 1 (SGLT1) is primarily expressed on the membrane of enterocytes, a type of epithelial cell found in the intestines, where it mediates the unidirectional absorption of glucose and galactose. Beyond its well-established role in nutrient absorption, SGLT1 also plays a protective role in maintaining the integrity of the intestinal barrier. Specifically, the natural ligand of SGLT1 (d-glucose) and a synthetic *C*-glucoside developed by our group can induce a protective anti-inflammatory effect on the intestinal epithelium. In this paper, we report the creation of a small library of *C*-glycoside, putative ligands for SGLT1, to gain further insights into its unclear mechanism of action. Preliminary biological experiments performed on an in vitro model of doxorubicin-induced mucositis, a severe intestinal inflammatory condition, indicate that the aromatic moiety present in all the compounds of the library is crucial for biological activity, while the sugar component appears to have less influence. These findings will be exploited to develop new, more potent anti-inflammatory compounds and to better understand and rationalize the protective mechanism of action.

## 1. Introduction

Sodium–glucose co-transporter 1 (SGLT1) is a high-affinity/low-capacity glucose and galactose transporter, mainly expressed on the apical membrane of intestinal epithelium cells and of cells of the S3 segment of the proximal tubule of kidneys [1,2,3,4]. SGLT1 is responsible for the unidirectional glucose and galactose absorption across the brush border membrane in the intestine [2,3,5], for sugar reuptake in kidneys, and for glucose uptake across the blood–brain barrier (BBB) [3]. Its fundamental physiological function has made this protein an important molecular target for the development of compounds able to inhibit sugar absorption, to be exploited as potential antidiabetic drugs [6,7,8,9], as well as compounds able to treat cardiovascular disorders [1,10,11].

**Figure 1:**
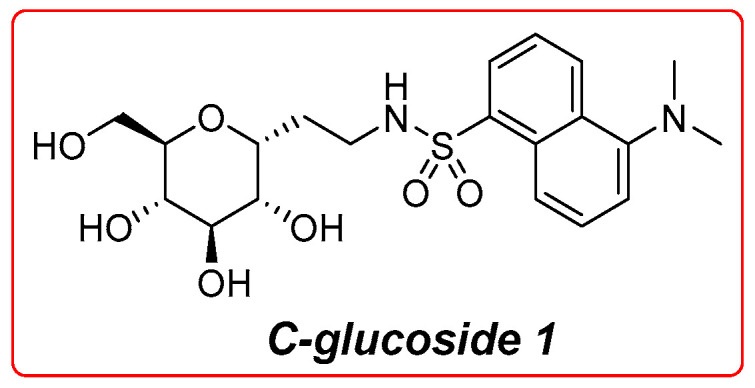
Structure of *C*-glucoside **1**.

SGLT1 plays a crucial role in the intestinal barrier by facilitating glucose absorption in the small intestine. Recent research has highlighted its importance not only in nutrient absorption but also in maintaining gut health. Studies have shown that SGLT1 influences gut microbiota composition and intestinal permeability, which are vital for overall metabolic health [12,13,14]. Additionally, the interaction of SGLT1 with other glucose transporters like GLUT2 is essential for efficient glucose uptake and regulation [5]. Emerging evidence suggests that SGLT1 inhibitors could modulate these processes, offering potential therapeutic avenues for metabolic disorders [15,16,17]. The crucial physiological function of this transporter reflects its wide expression in many tissues [18]. Significant efforts have been made to elucidate the physiological, functional, and structural features of this transporter; currently, the knowledge of its functional behavior is profound. Alongside the established physiological function, during the last decades, a new immunological role ascribable to this protein has been discovered; in fact, several works indicate its involvement in the d-glucose-mediated protection of the intestinal mucosa against a series of injuries caused by pathogens expressing lipopolysaccharides (LPSs) [19,20,21,22]. In our previous work, we observed that high glucose concentrations and a set of glycomimetics, tested both in vitro and orally on mice, were able to protect the intestinal epithelium from LPS-induced inflammatory injury in the murine models of septic shock, and data suggest an SGLT1 engagement in this phenomenon [23,24]. Among the synthesized glycomimetics, the most active was found to be compound **1** (Figure 1) which exerts a high protective effect at a concentration of several orders of magnitude lower than d-glucose [24]. This compound is a *C*-glycoside, a carbohydrate derivative where a carbon atom replaces the anomeric oxygen. This substitution greatly improves the chemical and metabolic stability of these compounds [25]. Glycoderivatives and glycomimetics, derived from carbohydrates, have attracted growing interest in drug development. They are promising candidates for anti-diabetic, antiviral, and antitumor therapies [26,27,28,29,30,31,32,33,34,35,36,37]. Their significance in drug discovery stems from their capacity to overcome the limitations of natural carbohydrates, including low stability under physiological conditions and suboptimal drug-like properties. In our previous research, we observed a significant protective role of *C*-glucoside **1** engagement towards intestinal mucosa in chemotherapy-induced mucositis [38]. Mucositis is a common pathological state and a serious side effect in patients treated with doxorubicin and 5-fluorouracil-based chemotherapy regimen for head and neck cancer and it derives from the direct cytotoxic effect of the drugs on the mucosal cells. The anticancer agents induce apoptosis and reduced proliferation in epithelial cells, leading to enhanced permeability and ulceration of the mucosae. For these reasons, it is necessary to develop new supporting therapies for chemotherapy-induced mucositis. Furthermore, we recently reported the preparation of a set of gold nanoparticles (AuNPs) decorated with the derivatives of d-glucose and *C*-glucoside **1** [39]. These AuNPs act as chemical tools to assess and evaluate the multivalent activation of SGLT1, as supported by the existing literature [40,41,42,43].

Our research has focused on understanding the protective role of glucose and of synthesized glycomimetics. In addition to studying potential intracellular processes, we are working on constructing a signal transduction pathway that connects SGLT1 activation to the blockage of the inflammatory response. Our efforts are also aimed at investigating how d-glucose and *C*-glycosides can activate SGLT1, involving it in cellular protection events. After creating a library of potential SGLT1 ligands [24], which mainly differ in the aromatic moiety, we decided to build a new small library of *C*-glucoside **1** analogs to explore the role of the sugar portion in the compounds’ activity [44]. The synthesized compounds have undergone preliminary testing for their protective activity in in vitro models of doxorubicin-induced mucositis [32] to gather initial information about the structure-activity relationship.

## 2. Results and Discussion

**Figure 2:**
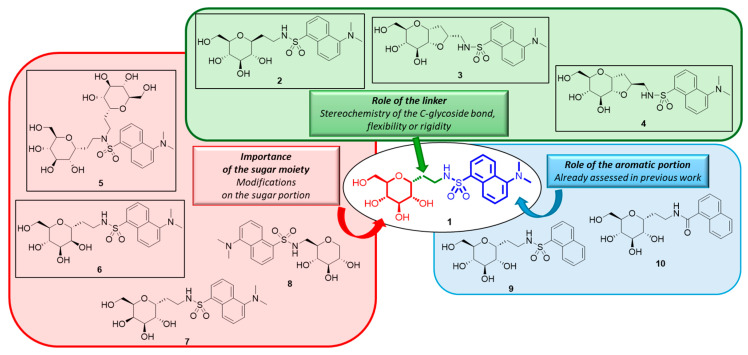
Structure of *C*-glucoside **1** analogs constituting the library object of this work. Newly synthesized compounds are black-circled.

Compound **1** (Figure 1) is a *C*-glucoside with a dansyl residue connected to a glucopyranose ring through an ethyleneamine spacer. The synthetic approach developed for this compound was previously described [24]. The biological evaluation of the small glycomimetic library prepared in that work aimed to identify the structural requirements correlated with anti-inflammatory activity on HT29 cell lines treated with LPS. The results indicated that the *C*-galactose analog is still effective as an anti-inflammatory agent, similar to compound **1**. The study also showed that the aromatic entity is crucial for the activity and that the absence of the dimethylamino group on the naphthyl moiety leads to a decrease in the anti-inflammatory effect. In this work, we shifted our focus to the importance of the sugar portion and the conformation/configuration of the spacer between the sugar part and the aromatic entity. In detail, we introduced rigidity through a second furanose cycle in compounds **3** and **4**, synthesized the mannose derivative **6**, and compound **5** bearing two sugar entities. These new analogs were tested for their anti-inflammatory effects in vitro alongside other *C*-glucoside **1** analogs (compounds **7**–**10**) previously synthesized and reported in our past work [24] (Figure 2). Specifically, new compounds **2**, **3**, and **4** represent the analogs of **1** with modifications on the linker/spacer between the sugar and aromatic portion (green box), while compounds **5** and **6** have variations associated with the sugar region (red box). The analogs of compound **1** with different aromatic portions (compounds **9** and **10**) were the subject of our previous study [24].

**Scheme 1:**
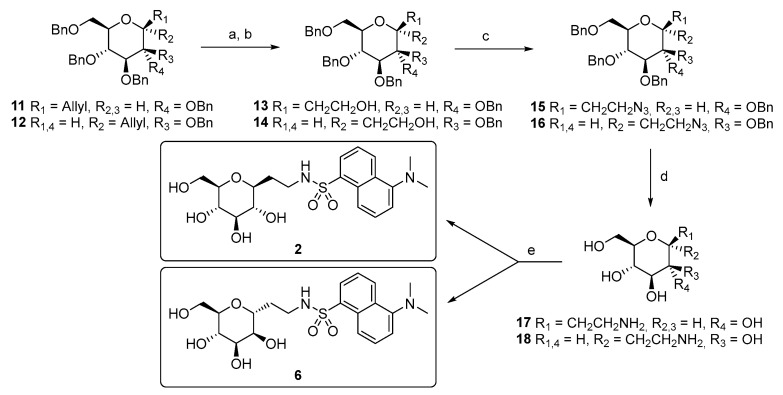
Synthesis of compounds **2** and **6**. Reagents and conditions: (a) O_3_, CH_2_Cl_2_, −78 °C, and 1 h, then Ph_3_P, −78 °C to r.t., and 12 h; (b) NaBH_4_, CH_2_Cl_2_/EtOH, r.t., 3 h, 66% for **13**, and 60% for **14** (two steps); (c) Ph_3_P, DIAD, (PhO)_2_PON_3_, THF dry, 0 °C to r.t., 1 h, 93% (**15**), and 80% (**16**); (d) H_2_, Pd(OH)_2_/C, AcOH, EtOAc/MeOH, r.t., and 24 h; (e) DansylCl, Et_3_N, H_2_O/THF, r.t., 3 h, 35% (**2,** over two steps), and 25% (**6**, over two steps).

Compound **2** is the β-glucoside analog of **1**. We planned to design and synthesize this compound because according to our hypothesis, β-glucosides should not have anti-inflammatory activity. This class of compounds acts as the inhibitors of SGLT1, rather than the activators/agonists of this transporter/receptor, as we believe compound 1 does. One example is the most important SGLT1 inhibitor, phlorizin, which binds to SGLT1, preventing glucose absorption mediated by the transporter. Furthermore, phlorizin can inhibit the glucose-mediated protective effect against inflammatory and apoptotic insults [21,22,23]. A similar approach was taken for compound **6**, the *manno*-analog. It is well established that d-mannose is neither recognized nor transported by SGLT1 [45]; therefore, assuming that our bioactive *C*-glycosides exert anti-inflammatory activity, as SGLT1 agonists, through a binding event to the same sugar interaction site, compound **6** should not have a protective effect. The synthetic pathways for compounds **2** and **6** are depicted in Scheme 1.

The synthesis starts from the known tetrabenzyl *C*-allyl glycoside precursors **11** [46,47] and **12** [48]. Through ozone-mediated oxidation and the subsequent hydride reduction, alcohols **13** and **14** were obtained, which were then transformed into the azido derivatives **15** and **16** using Mitzunobu reaction conditions. Hydrogenation/hydrogenolysis on the latter afforded unprotected *C*-ethyleneamine derivatives **17** and **18**, which were then converted into the final products **2** and **6** by reacting with dansyl chloride.

**Scheme 2:**
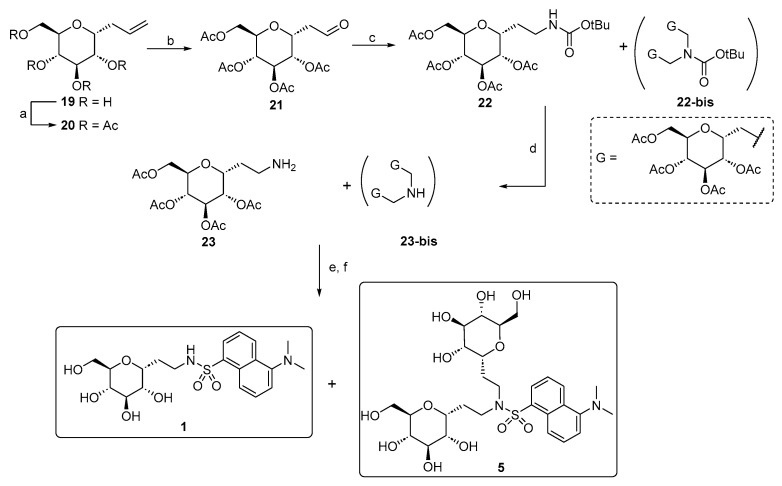
Synthesis of compounds 1 and 5. Reagents and conditions: (a) Ref. [47]; (b) OsO_4_, NaIO_4_, tBuOH/Acetone/H_2_O, r.t., and 5 h [49]; (c) tert-butylcarbamate, Et_3_SiH, TFA, CH_3_CN, r.t., and 3 h; (d) TFA 50% in CH_2_Cl_2_, r.t., and 2 h; (e) dansyl chloride, Et_3_N, MeOH, r.t., and 3 h; (f) MeONa, MeOH, r.t., and 2 h.

Compound **5** is a side product that was isolated during the synthesis of *C*-glucoside **1** following the synthetic strategy shown in Scheme 2. We investigated an alternative pathway for the production of **1** on a large scale, in which the ethylene amino linker was created by introducing the carbamate-protected amino group through a modified version of the classical reductive amination on intermediate aldehyde **21**.

It is plausible that the formation of a glucosyl dimer (compound **22-bis**) occurs during this final reaction step, as it has been reported that the formation of di- and tri-alkylated species often occurs in such reactions.

Compounds **3** and **4** are the bicyclic derivatives of **1**, containing a terminal amino group used for the dansyl linkage. The preparation of these compounds was initially planned as part of the development of *C*-glucoside **1** analogs with improved water solubility. We observed the poor solubility of **1** in aqueous media (0.5 mg/mL, 1 mM) and aggregation phenomena over time at higher concentrations, which could affect the homogeneity of the aqueous solutions and ultimately the compound’s pharmacokinetics. This behavior may be attributed to an intrinsic amphiphilicity of **1**; indeed, the flexibility of the linker may favor both the stacking of the aromatic moieties and hydrophobic interactions between the glucose and aromatic rings [50]. Therefore, we hypothesized that the insertion of a bicyclic scaffold could provide higher structural rigidity, preventing the formation of aggregates, and increasing water solubility, which would positively impact the compound’s efficacy. Furthermore, the introduction of a more rigid bicyclic system, compared to the linear and more flexible structure of **1**, could have a significant impact (either positive or negative) on the binding ability towards the putative SGLT1 binding site for these glycoderivatives. This binding is crucial for activation and ultimately for the anti-inflammatory and protective effect.

**Scheme 3:**
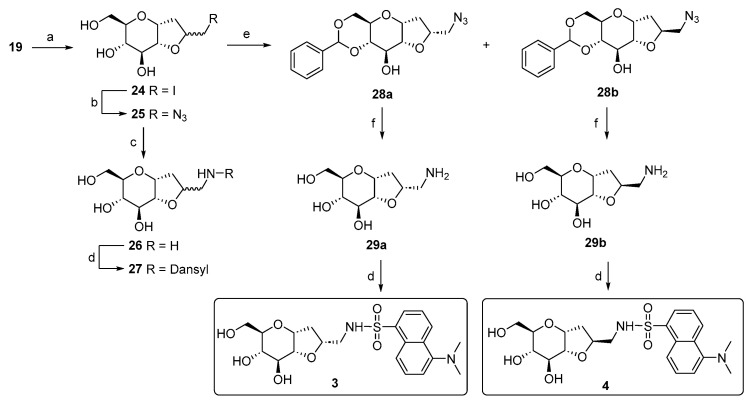
Synthesis scheme of bicyclic dansyl-*C*-glucosides **27**, **3,** and **4**. Reagents and conditions: (a) Ref. [48]; b) NaN_3_, DMF, 80 °C, and 24 h [51]; (c) Pd(OH)_2_/C, H_2_ atm., MeOH, r.t., and 12 h [51]; (d) dansyl chloride, Et_3_N, MeOH, r.t., 3 h [53], 50% for **27**, 41% for **3,** and 57% for **4**; (e) benzaldehyde dimethylacetal, CSA, dry DMF, 70 °C, 12 h, and 78% (51% for **28a** and 27% for **28b**); (f) Pd(OH)_2_/C, H_2_ atm., AcOH, r.t., and 24 h, quant.

The synthetic approach adopted for the preparation of the bicyclic variants has previously been developed by our group [51,52]. The principal transformation is the halocyclization reaction between the hydroxyl group at the C-2 position and the allylic double bond of α-*C*-allyl-glucopyranoside **19**, mediated by iodine-donating species, such as I_2_ or *N*-iodosuccinimide (NIS). This reaction generates an iodomethyl-2,7-dioxabicyclo[4.3.0]nonane scaffold, as a mixture of two diastereoisomers, depending on the side of attack of the 2-OH group on the iodonium intermediate. The resulting bicyclic *C*-glucoside structure bears an electrophilic site that has been exploited for a substitution reaction with sodium azide, generating the azido derivatives **25**, which can then be easily converted into the respective primary amines. During the synthesis, attempts to separate the two diastereoisomers using silica gel chromatography failed. Therefore, we completed the synthesis using **26** as a diastereoisomeric mixture, reacting it with dansyl chloride to produce **27** in a 7:3 ratio of the two diastereoisomers, as determined by a ^1^H-NMR analysis (Scheme 3).

**Figure 3:**
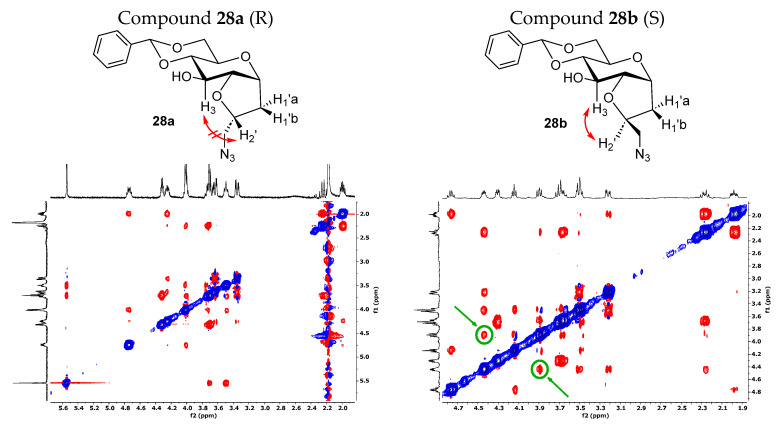
Two-dimensional ^1^H-NOESY experiments on compounds **28a** and **28b**. In the NOESY spectrum of **28b**, a cross peak between H2’ and H3 is present, indicating their spatial proximity. Higher-resolution 2D ^1^H-NOESY spectra are reported in Supplementary Materials.

We noticed a significantly higher water solubility for compound **27** compared to that of compound **1**. This result, along with the promising preliminary cellular assays (Figures 5 and 6), prompted us to plan and design a new synthetic route to obtain the pure diastereoisomeric forms of **27**. This was carried out to address two crucial aspects: (1) determining if the higher water solubility observed for **27** is an intrinsic behavior of the mixture or depends to a larger extent on one of the two diastereoisomers; (2) separating the two optical forms to discern their respective biological activity. In other words, we wanted to investigate if the biological activity relies on only one stereoisomer, or if the difference in stereochemistry is not crucial for the potential effect. Additionally, the resolution of the diastereoisomeric mixture allowed us to assign the correct stereochemistry of the newly formed chiral center. The separation of the two stereoisomers was achieved by adopting a strategy that had previously succeeded with the galactose analog [54]. Specifically, the mixture of the azido compound **25** was derivatized with a benzylidene acetal protecting group on the C4 and C6 positions of the bicyclic *C*-glucoside, using benzaldehyde dimethyl acetal in the presence of camphor sulfonic acid (CSA) as the catalyst. This facilitated an easy separation of the two stereoisomers through silica gel-flash chromatography. The subsequent reaction steps were carried out on the isolated diastereoisomers **28a** and **28b**. Firstly, a hydrogenolysis/hydrogenation reaction reduced the azide and simultaneously cleaved the acetal protecting group. Next, amines **29a** and **29b** were transformed into the respective sulphonamides by reacting with dansyl chloride. The final yields for compounds **3** and **4** (Scheme 3) reflect the diastereoisomeric excess of the iodocyclization reaction and thus the ratio between the two optical forms of 25. To assign the correct stereochemistry of the new chiral center, a 2D ^1^H-NOESY analysis was performed on the azido intermediates **28a** and **28b** (Figure 3). In the NOESY spectrum of **28b**, a clear cross peak between H2’ and H3 was observed, indicating their spatial proximity, which is characteristic of the diastereoisomer with an (*S*)-configuration at C2’. This cross peak was absent in the spectrum of the other diastereoisomer, as expected for the C2’ (*R*)-configuration, where the two protons are not in spatial proximity to generate a cross peak, corresponding to compound **28a**.

**Figure 4:**
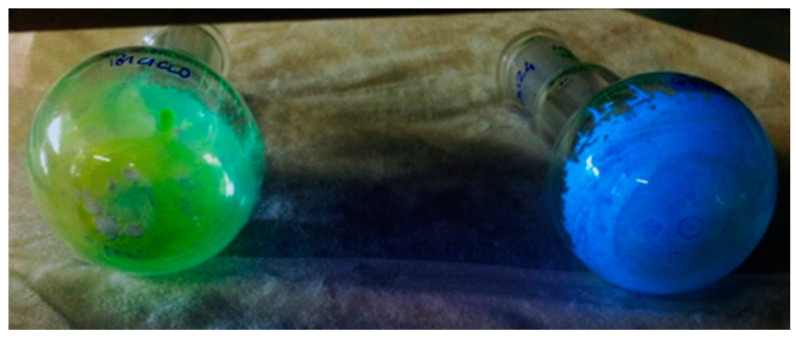
Optical behaviors of compounds **3** (on the **left**) and **4** (on the **right**) when exposed to a UVA lamp with an excitation wavelength of 365 nm.

The water solubility of the two final sulfonamide compounds was found to be completely different. Compound **3** was soluble at concentrations ranging from 0.5 to 5 mg/mL in water, while compound 4 was completely water-insoluble, even at concentrations lower than 0.5 mg/mL. Compound **27**, initially synthesized as a diastereoisomeric mixture of **3** and **4** in a 7:3 ratio, is well water-soluble within these concentration ranges, as 70% of it is composed of **3**. Surprisingly, we also observed a significant difference in fluorescence color between the two different optical forms (Figure 4) when the two compounds, in their solid form, were exposed to a UV lamp set at 365 nm. These observations are consistent with the differences in the physical and chemical characteristics that distinguish the two diastereoisomeric forms of the same molecule, as defined.

**Figure 5:**
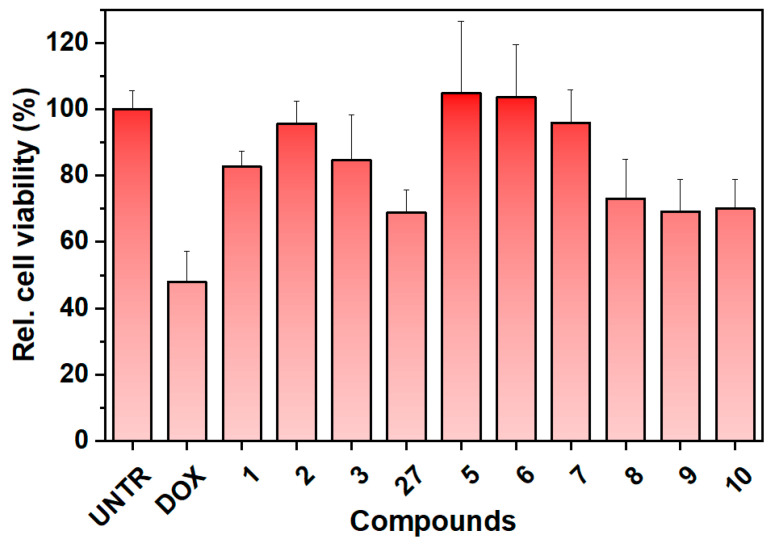
Preliminary viability experiments with the synthesized compounds. Neutral red assay for protective effect on doxorubicin-induced cytotoxicity on Caco-2 cells overexpressing SGLT1. The compounds were tested at 5 µg/mL, (8–10 µM). UNTR: untreated cells; DOX: cells treated with doxorubicin. The results are expressed as relative cell viability (%), as a percentage relative to the untreated cells.

Newly synthesized compounds **2–6**, compound **27** (as a mixture), and previously synthesized compounds **1** and **7–10** [24] (Figure 2) were tested for their protective activity on an in vitro model of doxorubicin-induced cytotoxicity using the SGLT1-overexpressing Caco-2 cell line (Figure 5). Unfortunately, compound **4** was excluded from the assessment due to its poor solubility in the water medium.

The preliminary results shown in Figure 5 indicate that the presence of the aromatic moiety (such as dansyl or naphthyl residues) is crucial for the protective activity of the tested compounds on cells. The sugar portion seems to have less influence, as cytoprotection is observed at 5 µg/mL for almost all the compounds comprising the β-glucoside and the *manno*-derivatives, for which we did not expect activity. The best cell viability results are associated with compounds **1** (*C*-glucoside **1**, the lead compound), **2** (β-*C*-glucoside analog), **3** ((*R*)-bicyclic derivative), **5** (dimer analog), **6** (*manno*-derivative), and **7** (*galacto*-derivative). Compounds **27** (diastereomeric mixture of the bicyclic derivative), **8, 9**, and **10** show a slightly reduced ability to preserve cell viability. The reduced activity of compound **27** can be attributed to the presence of the poorly soluble bicyclic derivative **4** in the mixture, which is primarily composed of the soluble portion associated with compound **3**. This affects the overall availability of the mixture and consequently the final biological effect. Structurally, compound **8** differs significantly from compound **1**, with the dansyl residue located on the amino group at C6 of a 1-deoxyglucoside, which may explain its lower activity. Compounds **9** and **10** lack the dimethylamino group on the naphthyl ring, which is a key functional group for binding and activity. This finding is consistent with our previous work [24]. The similar activity seen in compounds **2**, **3**, **5**, **6**, and **7** may be attributed to their higher structural similarity compared to **8**, **9**, and **10**. All these compounds are *C*-glycosides with a dansyl residue separated from the sugar ring by a two-carbon atom spacer. Using longer spacer chains reduces the protective activity [24]. The stereochemistry of the *C*-glycosidic bond and the configuration of the monosaccharide residue do not seem to be important for the overall biological efficacy. Despite the high specificity in monosaccharides recognition and translocation by SGLT1, other aspects must be considered.

**Figure 6:**
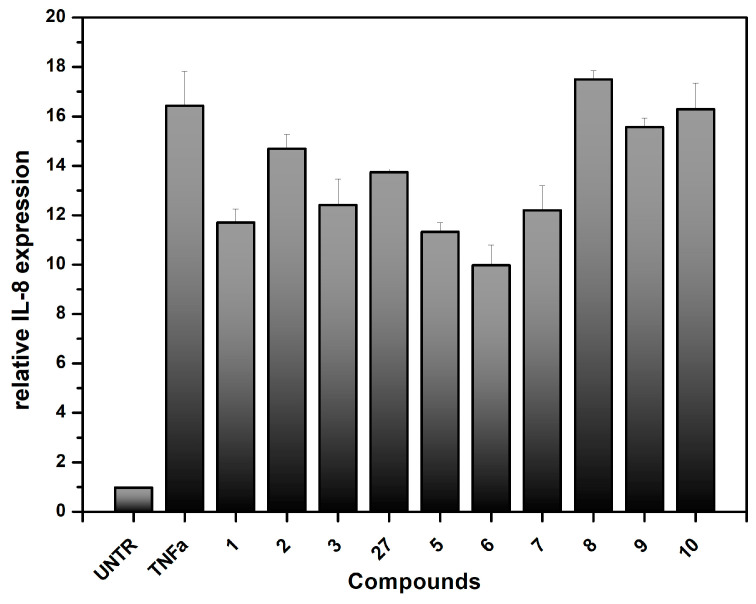
Effect of the synthesized compounds on IL-8 gene expression on the Caco-2 cell lines treated with TNFα as a pro-inflammatory cytokine. IL-8 gene expression was determined by real-time PCR. The compounds were tested at 5 µg/mL, (8–10 µM). UNTR: untreated cells; TNFα: Tumor necrosis factor-α. The results are expressed as relative IL-8 expression compared to the IL-8 expression on untreated cells.

Doxorubicin-induced cytotoxicity is associated with and results from severe inflammatory injury to intestinal epithelial cells, as reported in [38]. Severe inflammation leads to local damage of the intestinal mucosa, and several studies indicate the prominent role of the inflammation-inducer Tumor Necrosis Factor-α (TNF-α) in inducing inflammatory states [55,56,57]. TNF-α activates the transcription factors nuclear factor kappa B (NF-κB) and activator protein-1 (AP-1), which are key modulators of the inflammatory response [56]. Therefore, we decided to assess the ability of our synthesized compound to reduce the production of inflammatory markers in Caco-2 cells. One of the main pro-inflammatory cytokines produced by the Caco-2 cell line in response to TNF-α stimulation is Interleukin-8 (IL-8) [23,24,38,56]. The Caco-2 cells were treated with TNF-α in the presence or absence of the synthesized compounds. The mRNA levels of IL-8 produced by the cells were then measured using a qPCR protocol (Figure 6) [58,59]. Similar to the observations in the cytotoxicity experiments, compounds **2**–**7** and mixture **27** showed higher level of the inhibition of relative IL-8 expression compared to compounds **8**–**10**. The *C*-glycosidic framework and the presence of the dimethylamino group on the aromatic region are confirmed to be important for binding to SGLT1 and the resulting anti-inflammatory activity. However, it should be noted that no significant differences were observed among the *C*-glycosidic compounds. Additionally, the high level of IL-8 induction by TNF-α (TNFa bar in Figure 6), which is more than 15-fold compared to untreated cells (UNTR), must be considered. Considering the performed experiments, these preliminary results suggest that these compounds deserve significant attention. They also indicate that further investigations are needed to unravel their effective interaction with the transporter.

Various hypotheses have been proposed to describe the molecular recognition and translocation events involving SGLT1. Wright and Kinne suggested the existence of multiple sugar-binding sites in SGLT1, with one site being less stereospecific for sugar recognition [60,61,62,63,64]. It is hypothesized that the binding site for the synthesized *C*-glycosides in SGLT1 may overlap with the binding site involved in sugar recognition. For compounds **2**, **3**, **5**, **6**, and **7**, which contain the crucial dansyl residue, this ensures the highest level of recognition, targeting an unknown portion of the SGLT1 binding site. The presence of the sugar portion and the correct distance between the two regions allow for non- or less-stereospecific recognition by the same binding site region involved in sugar recognition.

## 3. Materials and Methods

### 3.1. Cell Cultures and Treatments

Cell lines Caco-2/bbe and Caco-2/bbe permanently transfected with SGLT-1 (kind gift of Prof. Mark Donowitz, MD, Johns Hopkins University School of Medicine, Baltimore) were cultured in DMEM high-glucose medium (4.5 g/L) (Euroclone, Pero, Italy) supplemented with 10% FBS (Euroclone), 1% glutamine (Euroclone), and 15 mM sterile HEPES solution (Euroclone) at 37 °C. For Caco-2/bbe/SGLT1 cells, 250 µg/mL G418 gentamicin bisulfate salt solution (Sigma-Aldrich, St. Louis, MO, USA) was added as an antibiotic agent; for normal Caco-2/bbe, a penicillin/streptamicin solution (Euroclone) was added. For viability assays, 1 × 10^5^ Caco-2 cells were plated on 96 flat-bottom well plates and treated after 24 h with doxorubicin (100 µM) with the compounds at concentrations of 5 µg/mL (8–10 µM). The cells were cultured at 37 °C and their viability was evaluated after 48 h with a Neutral Red assay kit (Merck©, Darmstadt, Germany). The absorbance of the extracted dye was measured using a plate reader at a wavelength of 540 nm according to the datasheet. For IL-8 gene expression experiments, total RNA was extracted from the compound-treated or untreated cell line and then processed as reported in [58,59]. The experimental data of the biological activity tests were processed and analyzed using Origin Pro, Version 2023b (OriginLab Corporation, Northampton, MA, USA).

### 3.2. Synthesis of Compounds

General remarks. All the commercial chemicals were purchased from Merck© (Darmstadt, Germany). All the chemicals were used without further purification. All the required anhydrous solvents were dried with molecular sieves for at least 24h prior to use. Thin layer chromatography (TLC) was performed on silica gel 60 F_254_ plates (Merck©, Darmstadt, Germany) with detection under UV light when possible, or by charring with a solution of (NH_4_)_6_Mo_7_O_24_ (21g), Ce(SO_4_)_2_ (1 g), concentrated H_2_SO_4_ (31 mL) in water (500 mL), or with an ethanol solution of ninhydrin or with Dragendorff spray reagent. Flash-column chromatography was performed on silica gel 230–400 mesh (Merck©, Darmstadt, Germany) or using the Isolera Flash Chromatography System (Biotage Sweden AB™, Uppsala, Sweden). ^1^H and ^13^C NMR spectra were recorded at 25 °C, unless otherwise stated, with a Varian Mercury 400 MHz instrument (Varian Inc., Palo Alto, CA, USA). Chemical shift assignments, reported in parts per million, were referenced to the corresponding solvent peaks. Mass spectra were recorded on an ABSciex 2000 QTRAP LC/MS/MS system with an ESI source (ABSciex©, Framingham, MA, USA).

2-(2,3,4,6-tetra-O-benzyl-β-d-glucopyranosyl)-ethanol **13**

In a 50 mL CH_2_Cl_2_ solution of **11** (1.42 g, 2.51 mmol) at −78 °C, O_3_ was bubbled until a pale blue color appeared, indicating the end of the reaction. The reaction was followed by TLC (petroleum ether/EtOAc 8:2). After 1 h, the excess of O_3_ was removed by purging the reaction with a stream of argon at −78 °C and then Ph_3_P (6.15 mmol, 2.45 eq) was added. The reaction was stirred for 12 h at r.t. Ph_3_P oxide was removed from the crude through a rapid filtration on a plug of silica gel using petroleum ether/EtOAc 7:3 as an eluent mixture. A ^1^H-NMR performed on the crude confirmed the complete transformation of the allyl group into an aldehyde function. A total of 736 mg of crude aldehyde (1.3 mmol) was dissolved in a mixture of CH_2_Cl_2_/MeOH 1:1 (6 mL, 0.2 M) at r.t. NaBH_4_ (5.19 mmol, 4 eq) was added and the reaction was stirred vigorously at r.t. The reaction was followed by TLC (petroleum ether/EtOAc 7:3). At the end of the reaction, the solvent was evaporated; the residue was suspended in a volume of a saturated solution of sodium carbonate and stirred vigorously for 30 min. The aqueous phase was extracted with EtOAc (3×). The organic phases were combined, dried with sodium sulfate, and filtrated. The product was purified from the residue by flash chromatography (petroleum ether/EtOAc 7:3) to provide compound **13** (646 mg, 1136 mmol, 66% over two steps).

Data for aldehyde intermediate (crude material): ^1^H NMR (400 MHz, CDCl_3_) δ 9.72 (s, 1H, CHO), 7.40–7.03 (m, 20H, CH Ar), 4.98–4.86 (m, 3H, CH_2_Ph), 4.82 (d, *J* = 10.7 Hz, 1H, CH_2_Ph), 4.63–4.55 (m, 3H, CH_2_Ph), 4.51 (d, *J* = 12.2 Hz, 1H, CH_2_Ph), 3.83 (td, *J* = 9.1, 4.5 Hz, 1H, H1), 3.77–3.63 (m, 4H, H2, H4, H6a, H6b), 3.52–3.44 (m, 1H, H5), 3.34 (t, *J* = 9.1 Hz, 1H, H3), 2.77–2.67 (m, 1H, H1’a), 2.57 (ddd, *J* = 16.2, 7.9, 2.4 Hz, 1H, H1’b).

^13^C NMR (101 MHz, CDCl_3_) δ 200.44 (C2’ (CHO)), 138.47, 138.06, 137.69 (Cq Ar × 4), 129.79, 128.73, 128.68, 128.64, 128.58, 128.53, 128.34, 128.19, 128.05, 127.98, 127.92, 127.86, 127.83, 127.80 (C Ar × 20), 87.22, 81.25, 79.25, 78.39 (C1, C2, C3, C4, C5), 75.73, 75.21 (CH_2_Ph × 4), 74.58 (C5), 68.75 (C6), 46.20 (C1’).

C_36_H_38_O_6_; calcd. mass 566.69; ESI-MS: *m*/*z* 567.61 [M + H]^+^.

Data for compound **13**. ^1^H NMR (400 MHz, CDCl_3_) δ 7.38–7.23 (m, 18H, CH Ar), 7.23–7.13 (m, 2H, CH Ar), 4.93–4.87 (m, 3H, CH_2_Ph), 4.83 (d, *J* = 10.8 Hz, 1H, CH_2_Ph), 4.64 (d, *J* = 10.9 Hz, 1H, CH_2_Ph), 4.59–4.49 (m, 3H, CH_2_Ph), 3.79 (t, *J* = 5.4 Hz, 2H, H2’a,b), 3.73–3.64 (m, 2H, H6a, H3), 3.63–3.54 (m, 2H, H6b, H2), 3.54–3.44 (m, 2H, H1, H5), 3.35 (t, *J* = 9.2 Hz, 1H, H4), 2.12–2.00 (m, 1H, H1’a), 1.84–1.67 (m, 1H, H1’b).

^13^C NMR (101 MHz, CDCl_3_) δ 138.55, 138.02, 137.97, 137.95 (Cq Ar), 128.63, 128.61, 128.58, 128.56, 128.17, 128.11, 128.08, 127.99, 127.96, 127.86, 127.83 (C Ar × 20), 87.11 (C3), 81.85 (C2), 80.00 (C1), 78.72 (C4), 78.57 (C5), 75.75, 75.47, 75.21, 73.60 (CH_2_Ph), 69.17 (C6), 61.68 (C2’), 33.80 (C1’).

C_36_H_40_O_6_; calcd. mass 568.61; ESI-MS: *m*/*z* 569.64 [M + H]^+^.

2-(2,3,4,6-tetra-O-benzyl-α-d-mannopyranosyl)-ethanol **14**

A solution of compound **12** (2,27 g, 4 mmol) in dry CH_2_Cl_2_ (30 mL, ≈0.1 M) was cooled to −78 °C, and ozone was bubbled through the solution for approximately 3 h until the solution turned blue. The ozone was then removed from the reaction by bubbling argon through the solution and Ph_3_P (3.3 g, 12.5 mmol, 3 eq) was added at −78 °C. The solution was slowly warmed to r.t. and stirred overnight. The reaction was followed by TLC (petroleum ether/EtOAc 8:2). The crude product was passed through a plug of silica gel, as reported for compound **13**. A ^1^H-NMR performed on the crude confirmed the complete transformation of the allyl group into an aldehyde function. The crude aldehyde was dissolved in a mixture of CH_2_Cl_2_/MeOH 1:1 (80 mL, ≈0.05 M). NaBH_4_ (605 mg, 16 mmol, 4 eq related to compound **12**) was added at r.t. and the reaction was stirred at r.t. for 2 h. The product was extracted as for compound **13** and purified by flash chromatography (petroleum ether/EtOAc 6:4). A total of 1363 mg (2.4 mmol, 60% yield over two steps) of alcohol **14** were obtained.

^1^H NMR (400 MHz, CDCl_3_) δ 7.39–7.23 (m, 18H, CH Ar), 7.23–7.16 (m, 2H, CH Ar), 4.63–4.45 (m, 8H, CH_2_Ph), 4.21–4.13 (m, 1H, H1), 4.01–3.94 (m, 1H, H5), 3.84–3.74 (m, 4H, H3, H6a, H2’a,b), 3.71 (t, *J* = 5.7 Hz, 1H, H4), 3.66–3.55 (m, 2H, H2, H6b), 2.71 (s, 1H, OH), 1.93–1.81 (m, 1H, H1’a), 1.80–1.71 (m, 1H, H1’b).

^13^C NMR (101 MHz, CDCl_3_) δ 138.13, 138.11, 137.69, 137.66 (Cq Ar), 129.84, 128.56, 128.53, 128.52, 128.47, 128.11, 128.00, 127.92, 127.91, 127.88, 127.77 (C Ar), 76.29 (C2), 76.00 (C3), 74.91 (C4), 73.63 (C5), 73.40 (CH_2_Ph), 72.49 (CH_2_Ph), 72.25 (C1), 71.74 (CH_2_Ph), 68.68 (C6), 61.56 (C2’), 32.25 (C1’).

C_36_H_40_O_6_; calcd. mass 568.71; ESI-MS: *m*/*z* 569.6 [M + H]^+^, 591.5 [M + Na]^+^.

2-(2,3,4,6-tetra-O-benzyl-β-d-glucopyranosyl)-1-azidoethane **15**

Compound **13** (631 mg, 1.1 mmol) was dissolved in dry THF (5.5 mL, 0.2 M) and Ph_3_P (3.3 mmol, 3 eq) was added. The solution was cooled to 0 °C and DIAD (3.3 mmol, 3 eq) was added dropwise. After 10 min, 3.5 mmol (3.2 eq) of diphenylphosphoryl azide (PhO)_2_P(O)N_3_ was added at 0 °C and the reaction was stirred for 1 h at r.t. The reaction was followed by TLC (petroleum ether/EtOAc 8:2) and the product was purified from the crude by flash chromatography (eluent petroleum ether/EtOAc 9:1) to provide compound **15** (621 mg, 1.03 mmol, 93%).

^1^H NMR (400 MHz, CDCl_3_) δ 7.38–7.23 (m, 18H CH Ar), 7.20–7.13 (m, 2H CH Ar), 4.98–4.87 (m, 3H, CH_2_Ph), 4.84 (d, *J* = 10.8 Hz, 1H, CH_2_Ph), 4.69–4.61 (m, 2H, CH_2_Ph), 4.58 (d, *J* = 10.8 Hz, 1H, CH_2_Ph), 4.54 (d, *J* = 12.2 Hz, 1H, CH_2_Ph), 3.76–3.64 (m, 4H, H2, H3, H4, H6a), 3.45–3.37 (m, 3H, H1,H5, H6b), 3.35 (dd, *J* = 9.2, 2.3 Hz, 1H, H2’a), 3.33–3.25 (m, 1H, H2’b), 2.17–2.04 (m, 1H, H1’a), 1.75–1.63 (m, 1H, H1’b).

^13^C NMR (101 MHz, CDCl_3_) δ 138.63, 138.23, 138.19, 138.00 (Cq Ar), 128.66, 128.61, 128.56, 128.52, 128.22, 128.09, 128.03, 127.95, 127.90, 127.87, 127.82 (C Ar), 87.36 (C3), 81.94 (C2), 78.93 (C1), 78.48 (C4), 76.39 (C5), 75.74, 75.39, 75.13, 73.62 (CH_2_Ph), 68.95 (C6), 48.04 (C2’), 29.85 (C1’).

C_36_H_39_N_3_O_5_; calcd. mass 593.72; ESI-MS: *m*/*z* 594.68 [M + H]^+^.

2-(2,3,4,6-tetra-O-benzyl-α-d-mannopyranosyl)-1-azidoethane **16**

Compound **14** (653 mg, 1.148 mmol) was converted into azido derivative **16** following the same procedure for compound **15**. The crude product was purified by flash chromatography (eluent petroleum ether/EtOAc 9:1) to afford 549 mg (0.925 mmol, 80%) of azido derivative **16**.

^1^H NMR (400 MHz, CDCl_3_) δ 7.39–7.19 (m, 20H CH Ar), 4.63 (d, *J* = 11.7 Hz, 1H, CH Ar), 4.60–4.50 (m, 7H, CH Ar), 4.09–4.02 (m, 1H, H1), 3.91 (dd, *J* = 10.6, 4.8 Hz, 1H, H5), 3.86–3.77 (m, 3H, H3, H6a, H4), 3.73 (dd, *J* = 10.1, 4.6 Hz, 1H, H6b), 3.58 (dd, *J* = 6.3, 2.8 Hz, 1H, H2), 3.49–3.32 (m, 2H, H2’a,b), 1.93–1.73 (m, 2H, H1’a,b).

^13^C NMR (101 MHz, CDCl_3_) δ 138.35, 138.14, 138.07, 138.02 (Cq Ar), 128.52, 128.50, 128.45, 128.12, 128.03, 128.00, 127.88, 127.81, 127.68 (C Ar), 75.95 (C2), 75.55 (C3), 74.54 (C4), 74.05 (C5), 73.40 (CH_2_Ph), 73.27 (CH_2_Ph), 72.36 (CH_2_Ph), 71.52 (CH_2_Ph), 68.89 (C1), 68.76 (C6), 48.06 (C2’), 29.93 (C1’).

C_36_H_39_N_3_O_5_; calcd. mass 593.72; ESI-MS: *m*/*z* 594.6 [M + H]^+^.

2-(β-d-glucopyranosyl)-ethanamine **17**

Azide **15** (610 mg, 1.026 mmol) was dissolved in a mixture of EtOAc/MeOH and the solution was degassed under vacuum for 10 min. A catalytic amount of Pd(OH)_2_/C and 0.1 eq of AcOH were added and the reaction was stirred under a H_2_ atmosphere for 48 h. The reaction was followed by TLC (petroleum ether/EtOAc 8:2 and CH_2_Cl_2_/MeOH/NH_3_ 5:5:1) until the disappearance of the starting compound. The reaction was filtered through a celite pad, concentrated, and used directly for the next reaction.

^1^H NMR on crude material (400 MHz, D_2_O) δ 3.89–3.75 (m, 1H, H6a), 3.63 (dt, *J* = 12.1, 4.5 Hz, 1H, H5, H6b), 3.34 (ddt, *J* = 18.4, 12.7, 8.3 Hz, 4H, H1, H2, H3, H4), 3.25–3.05 (m, 3H, H2’a,b, H5), 2.25–2.07 (m, 1H, H1’a), 1.84–1.69 (m, 1H, H1’b).

C_8_H_17_NO_5_; calcd. mass 207.23; ESI-MS: *m*/*z* 208.20 [M + H]^+^.

2-(α-d-mannopyranosyl)-ethanamine **18**

Compound **16** (493 mg, 0.83 mmol) was dissolved in a mixture of EtOAc/MeOH and the solution was degassed under vacuum for 10 min. A catalytic amount of Pd(OH)_2_/C and 0.1 eq of AcOH were added and the reaction was stirred under a H_2_ atmosphere for 72 h. The reaction was followed by TLC (petroleum ether/EtOAc 9:1 and EtOAc/MeOH/H_2_O/AcOH 5:5:1:1) until the disappearance of the starting compound. The reaction was filtered through a celite pad, concentrated, and used directly for the next reaction.

^1^H NMR on crude compound (400 MHz, D_2_O) δ 4.03–3.95 (m, 1H, H6a), 3.87–3.75 (m, 3H, H6b, H3, H2), 3.70 (dd, *J* = 12.1, 6.8 Hz, 1H, H1), 3.60 (t, *J* = 8.9 Hz, 1H, H4), 3.55–3.46 (m, 1H, H5), 3.18–3.00 (m, 2H, H2’), 2.23–2.08 (m, 1H, H1’a), 1.95–1.77 (m, 1H, H1’b).

C_8_H_17_NO_5_; calcd. mass 207.23; ESI-MS: *m*/*z* 208.23 [M + H]^+^.

5-(N,N-Dimethylamino)-N-[2-(β-d-glucopyranosyl)ethyl]-1-naphthalensulfonamide **2**

To a solution of crude compound **17** (225 mg) in a mixture of H_2_O/THF 1:1 (10 mL, 0.1 M), 285 µL of Et_3_N (2.05 mmol, 2 eq) was added at r.t. After 10 min, 410 mg (1.53 mmol, 1.5 eq) of dansyl chloride was added and the reaction was stirred at r.t. for 3 h. The reaction was followed by TLC (CH_2_Cl_2_/MeOH/NH_3_ 5:5:1 and EtOAc/MeOH 9:1) and the product was purified by flash chromatography (eluent EtOAc/MeOH 9.5:0.5). A total of 154 mg (0.36 mmol, 35% over two steps) of compound **2** were obtained.

^1^H NMR (400 MHz, CD_3_OD) δ 8.56 (d, *J* = 8.5 Hz, 1H, CH Ar), 8.36 (d, *J* = 8.7 Hz, 1H, CH Ar), 8.20 (d, *J* = 7.3 Hz, 1H, CH Ar), 7.64–7.51 (m, 2H, CH Ar), 7.27 (d, *J* = 7.5 Hz, 1H, CH Ar), 3.62 (dd, *J* = 11.8, 2.2 Hz, 1H, H6a), 3.50 (dd, *J* = 11.8, 5.5 Hz, 1H, H6b), 3.21–3.09 (m, 2H, H4, H3), 3.09–2.96 (m, 3H, H1, H2’a,b), 2.95–2.79 (m, 8H, (CH_3_)_2_N-, H5, H2), 2.04–1.91 (m, 1H, H1’a), 1.50–1.37 (m, 1H, H1’b).

^13^C NMR (101 MHz, CD_3_OD) δ 153.20 (Cq Ar), 136.78 (Cq Ar), 131.25 (Cq Ar), 131.13 (C Ar), 130.94 (Cq Ar), 130.34 (C Ar), 129.08 (C Ar), 124.30 (C Ar), 120.66 (C Ar), 116.45 (C Ar), 81.18 (H4), 79.64 (H3), 78.08 (C1), 75.26 (C2), 71.62 (C5), 62.78 (C6), 45.83 ((CH_3_)_2_N- x2), 40.58 (C2’), 32.90 (C1’).

C_10_H_28_N_2_O_7_S; calcd. mass 440.51; ESI-MS: *m*/*z* 441.6 [M + H]^+^. [α]_D_^20^ +75 (c = 0.5, MeOH).

5-(N,N-Dimethylamino)-N-[2-(α-d-mannopyranosyl)ethyl]-1-naphthalensulfonamide **6**

To a solution of crude compound **18** (186 mg) in MeOH (8 mL, 0.1 M), 230 µL of Et_3_N (1.66 mmol, 2 eq) was added at r.t. After 10 min, 330 mg (1.24 mmol, 1.5 eq) of dansyl chloride was added and the reaction was stirred at r.t. for 3 h. The reaction was followed by TLC (EtOAc/MeOH/H_2_O/AcOH 5:5:1:1 and EtOAc/MeOH 9:1) and the product was purified by flash chromatography (eluent EtOAc/MeOH 8.5:1.5). A total of 91 mg (0.2 mmol, 25% over two steps) of compound **6** were obtained.

^1^H NMR (400 MHz, CD_3_OD) δ 8.56 (d, *J* = 8.5 Hz, 1H, CH Ar), 8.34 (d, *J* = 8.7 Hz, 1H, CH Ar), 8.20 (d, *J* = 7.2 Hz, 1H, CH Ar), 7.63–7.54 (m, 2H, CH Ar), 7.27 (d, *J* = 7.5 Hz, 1H, CH Ar), 3.86–3.79 (m, 1H, H1), 3.71–3.64 (m, 2H, H6a,b), 3.58–3.45 (m, 3H, H2, H3, H4), 3.35 (m, 1H, H5), 2.95 (t, *J* = 6.9 Hz, 2H, H2’a,b), 2.88 (s, 6H, (CH_3_)_2_N-), 1.86–1.73 (m, 1H, H1’a), 1.62–1.48 (m, 1H, H1’b).

^13^C NMR (101 MHz, D_2_O) δ 150.57 (Cq Ar), 133.01 (Cq Ar), 130.21 (C Ar), 130.07 (C Ar), 128.87 (Cq Ar), 128.75 (Cq Ar), 128.64 (C Ar), 123.95 (C Ar), 119.28 (C Ar), 116.11 (C Ar), 78.57 (C5), 77.03 (C1), 75.35 (C2), 72.86 (C4), 68.96 (C3), 59.92 (C6), 44.86 ((CH_3_)_2_N-), 38.14 (C2’), 30.07 (C1’).

C_20_H_28_N_2_O_7_S; calcd. mass 440.51; ESI-MS: *m*/*z* 441.6 [M + H]^+^. [α]_D_^20^ +39 (c = 0.3, MeOH).

3-(2,3,4,6-tetra-O-acetyl-α-d-glucopyranosyl)-propene **20**

The synthesis and characterization of this compound are described in [47]. All the NMR data are in agreement with those published.

2-(2,3,4,6-tetra-O-acetyl-α-d-glucopyranosyl)-ethanal **21**

The synthesis and characterization of this compound are described in [49,65]. All the NMR results are consistent with the published data.

2-(2,3,4,6-tetra-O-acetyl-α-d-glucopyranosyl)-tert-butoxycarbonylaminoethane **22**

Compound **21** (800 mg, 2.14 mmol) was dissolved in dry CH_3_CN (10 mL) under an argon atmosphere. Tert-butyl carbamate (6.42 mmol, 3 eq.), triethylsilane (6.42 mmol, 3 eq.), and TFA (6.21 mmol, 2.9 eq) were successively added to the solution. The reaction was followed by TLC (petroleum ether/EtOAc 7:3), which was stirred at r.t. After 3 h, Et_3_N was added to neutralize the reaction and the solvent evaporated. The residue was directly used for the next reaction step.

^1^H NMR on crude **22** (400 MHz, CDCl_3_) δ 5.51 (d, *J* = 18.6 Hz, 1H, NH), 5.18 (dd, *J* = 18.9, 9.9 Hz, 1H, H1), 5.01–4.78 (m, 3H, H2, H3, H4), 4.27–4.12 (m, 2H, H4, H6a), 4.07–3.95 (m, 1H, H6b), 3.92–3.76 (m, 1H, H5), 3.24–3.00 (m, 1H), 2.05–1.92 (m, 13H), 1.65 (s, 1H), 1.35 (s, 9H).

^13^C NMR on crude **22** (101 MHz, CDCl_3_) δ δ 170.60, 169.91, 169.58, 169.48 (4× COCH_3_), 155.80 (NHCO), 79.23 (CH_3_C-O-tBu), 70.76, 70,70 69.89, 69.19, 68.44 (C1, C2, C3, C4, C5), 62.00, 61.81 (C6, C2’), 37.08 (C2’), 28.69, 28.35, 28.28, 28.22 (4x CH_3_CO-Ac), 25.78 (C1’), 20.64 (CH_3_C-O-tBu x 3).

C_21_H_33_NO_11_; calcd. mass 475.49; ESI-MS: *m*/*z* 476.5 [M + H]^+^, 498.5 [M + Na]^+^.

2-(2,3,4,6-tetra-O-acetyl-α-d-glucopyranosyl)-ethaneamine **23**

To a solution of crude **22** (1 g) in CH_2_Cl_2_ (7.5 mL), 7.5 mL of trifluoroacetic acid was added. The reaction was followed by TLC (EtOAc/MeOH/H_2_O/AcOH 7:3:1:1) and after 2 h the solvent was evaporated. Compound **23** was directly used for the next reaction without further purifications.

^1^H NMR on crude product (400 MHz, CDCl_3_) δ 5.15 (t, *J* = 8.9 Hz, 1H, H3), 4.91 (dd, *J* = 9.3, 5.6 Hz, 1H, H2), 4.83 (td, *J* = 8.9, 2.4 Hz, 1H, H4), 4.22–4.03 (m, 2H, H1, H6a), 3.94 (dd, *J* = 12.2, 2.7 Hz, 1H, H6b), 3.87–3.69 (m, 1H, H5), 2.79–2.52 (m, 2H, H2’), 2.01–1.86 (m, 13H, 3 x CH_3_CO, H1’a), 1.62–1.46 (m, 1H, H1’b).

^13^C NMR on crude product (101 MHz, CDCl_3_) δ 170.49, 169.85, 169.50, 169.38 (4x COCH_3_), 70.59, 69.94, 69.85, 68.94, 68.46 (C1, C2, C3, C4, C5), 62,04 (C6), 37.82 (C2’), 27.86 (C1’).

C_16_H_25_NO_9_; calcd. mass 375.37; ESI-MS: *m*/*z* 376.5 [M + H]^+^.

5-(N,N-Dimethylamino)-N,N-di-[2-(α-d-glucopyranosyl)ethyl]-1-naphthalensulfonamide **5**

Compound **5** was isolated as a by-product during gram-scale synthesis of **1** depicted in Scheme 2 (see Results and Discussion). A total of 1 g of crude amine **23** was dissolved in dry CH_2_Cl_2_ (10 mL). An excess of Et_3_N and dansyl chloride were then added and the reaction stirred at r.t. The reaction was followed by TLC (petroleum ether/EtOAc 6:4 and EtOAc/MeOH/AcOH 6:3:1). After 3 h, the reaction was concentrated and the residue was subjected to flash chromatography on silica gel (petroleum ether/EtOAc 6:4). A total of 0.54 g (0.55 mmol) of the peracetylated precursor of dimer **5** and 300 mg (0.49 mmol) of the monomer derivative (compound **1** in peracetylated form) were obtained, as confirmed by an ESI-MS analysis (*m*/*z* 967.3 [M + H]^+^ for the dimer and 609.2 [M + H]^+^ for the monomer). The dimer was directly deprotected with MeONa (6 mL of 1 M solution in MeOH) in MeOH and CH_2_Cl_2_ (10 + 5 mL). After 2 h, the pH was adjusted to neutrality adding IRA-120 H^+^ resin. After its removal by filtration, the filtrate was concentrated to obtain **5** with quantitative yield.

^1^H NMR (400 MHz, CD_3_OD) δ 8.56 (d, *J* = 8.4 Hz, 1H, CH Ar), 8.29 (d, *J* = 8.7 Hz, 1H, CH Ar), 8.21 (d, *J* = 7.3 Hz, 1H, CH Ar), 7.63–7.53 (m, 2H, CH Ar), 7.25 (d, *J* = 7.5 Hz, 1H, CH Ar), 3.94–3.81 (m, 2H, H1 (x2)), 3.75 (d, *J* = 11.7 Hz, 2H, H6a (x2)), 3.65–3.47 (m, 6H, H6b (x2), H2 (x2), H2’a (x2)), 3.41 (t, *J* = 9.0 Hz, 2H, H3 (x2)), 3.37–3.27 (m, 4H, H5 (x2), H2’b (x2)), 3.20 (t, *J* = 9.1 Hz, 2H, H4 (x2)), 2.87 (s, 6H, (CH_3_)_2_N-), 2.03–1.75 (m, 4H, H1’a,b (x2)).

^13^C NMR (101 MHz, CD_3_OD) δ 153.15 (Cq Ar), 136.13 (Cq Ar), 131.52 (C Ar), 131.32 (Cq Ar), 131.25 (Cq Ar), 130.91 (C Ar), 129.18 (C Ar), 124.38 (C Ar), 120.64 (C Ar), 116.45 (C Ar), 75.16 (C3), 74.88 (C1), 74.53 (C5), 72.62 C2), 72.12 (C4), 62.99 (C6), 45.82 ((CH_3_)_2_N-), 45.71 (C2’), 24.92 (C1’).

C_28_H_42_N_2_O_12_S; calcd. mass 630.71; ESI-MS: *m*/*z* 631.3 [M + H]^+^ 653.3 [M + Na]^+^. [α]_D_^20^ +122 (c = 0.7, MeOH).

Compounds **24**, **25**, **26**

The synthesis and characterization of this compound are described in [51]. All the NMR data are in agreement with those published.

Compound **27**

The synthesis and characterization of this compound are described in [53]. All the NMR data are in agreement with those published.

(2R,4aR,5aR,7R,8aR,9R,9aS)-7-(azidomethyl)-2-phenyloctahydrofuro[2′,3′:5,6]pyrano[3,2-d][1,3]dioxin-9-ol **28a** and (2R,4aR,5aR,7S,8aR,9R,9aS)-7-(azidomethyl)-2-phenyloctahydrofuro[2′,3′:5,6]pyrano[3,2-d][1,3]dioxin-9-ol **28b**

Compound **24** (mixture of diastereoisomers) (1115 mg, 4.54 mmol) was dissolved in dry DMF (5 mL, ≈1 M). Benzaldehyde dimethylacetal (5.45 mmol, 1.2 eq) and camphorsulphonic acid (2.27 mmol, 0.5 eq) were added at r.t. The reaction was stirred at 70 °C for 12 h, and followed by TLC (petroleum ether/EtOAc 2:8). Et_3_N (5 mL) was added to neutralize the acidity of the reaction and the solvent was evaporated. The crude was purified by flash chromatography (eluent petroleum ether/EtOAc 6:4 to petroleum ether/EtOAc 1:1), obtaining compound **28a** (777 mg, 2.33 mmol, 51% yield) and compound **28b** (405 mg, 1.21 mmol, 27% yield) with an overall yield of 78%.

Data for **28a**. ^1^H NMR (400 MHz, CDCl_3_) δ 7.50 (dd, *J* = 6.6, 2.9 Hz, 2H, CH Ar), 7.42–7.33 (m, 3H, CH Ar), 5.51 (s, 1H, H7), 4.66 (dt, *J* = 11.1, 7.0 Hz, 1H, H1), 4.32–4.22 (m, 1H, H6a), 4.16 (td, *J* = 9.9, 4.1 Hz, 1H, H2’), 4.00–3.87 (m, 2H, H2, H3), 3.71–3.63 (m, 2H, H5, H6b), 3.55 (dd, *J* = 13.0, 3.8 Hz, 1H, H3’a), 3.44 (dd, *J* = 11.6, 6.6 Hz, 1H, H4), 3.28 (dd, *J* = 13.0, 4.2 Hz, 1H, H3’b), 2.14 (dd, *J* = 22.3, 11.1 Hz, 1H, H1’a), 1.98–1.86 (m, 1H, H1’b).

^13^C NMR (101 MHz, CDCl_3_) δ 137.08 (CqAr), 129.25 (CHAr), 128.33 (CHAr), 126.31 (CHAr), 101.77 (CHPh), 79.84 (CH), 79.11 (CH), 75.94 (CH), 75.87 (CH), 73.36 (CH), 68.98 (CH_2_), 64.00 (CH), 54.14 (CH_2_), 29.71 (CH_2_).

C_16_H_19_N_3_O_5_; calcd. mass 333.34; ESI-MS: *m*/*z* 334.4 [M + H]^+^.

Data for **28b**. ^1^H NMR (400 MHz, CDCl_3_) δ 7.49 (dd, *J* = 6.5, 3.0 Hz, 2H, CH Ar), 7.42–7.32 (m, 3H, CH Ar), 5.52 (s, 1H, H7), 4.73 (dd, *J* = 15.9, 8.9 Hz, 1H, H1), 4.39 (dq, *J* = 8.3, 4.1 Hz, 1H, H2’), 4.29 (dd, *J* = 9.6, 4.1 Hz, 1H, H6a), 4.11 (t, *J* = 7.1 Hz, 1H, H2), 3.84 (dd, *J* = 9.6, 7.3 Hz, 1H, H3), 3.68 (t, *J* = 9.8 Hz, 1H, H6b), 3.65–3.58 (m, 1H, H5), 3.52–3.39 (m, 2H, H4, H3’a), 3.18 (dd, *J* = 12.9, 4.2 Hz, 1H, H3’b), 2.22 (dt, *J* = 13.2, 9.1 Hz, 1H, H1’a), 1.94 (ddd, *J* = 13.3, 9.0, 4.5 Hz, 1H, H1’b).

^13^C NMR (101 MHz, CDCl_3_) δ 137.08 (Cq Ar), 129.39 (CH Ar), 128.45 (CH Ar), 126.35 (CH Ar), 101.89 (CHPh), 80.16 (CH), 79.97 (CH), 75.46 (CH), 75.30 (CH), 71.02 (CH), 69.09 (CH_2_), 63.83 (CH), 54.93 (CH_2_), 29.46 (CH_2_).

C_16_H_19_N_3_O_5_; calcd. mass 333.34; ESI-MS: *m*/*z* 334.4 [M + H]^+^.

(2R,3aR,5R,6S,7S,7aR)-2-(aminomethyl)-5-(hydroxymethyl)hexahydro-2H-furo[3,2-b]pyran-6,7-diol **29a**

To a solution of compound **28a** (94 mg, 0.28 mmol) in a mixture of MeOH/EtOAc (5 + 3 mL) were added a few drops of glacial acetic acid and a catalytic amount of Pd(OH)_2_/C. The reaction was stirred at r.t. under a hydrogen atmosphere until TLC (petroleum ether/EtOAc 1:1 and EtOAc/MeOH/H_2_O/AcOH 6:4:1:1) indicated the disappearance of the starting material. After 24 h, the reaction was then filtered through a pad of celite and the solution containing the product was concentrated. ^1^H-NMR confirmed the purity of the deprotected bicyclic sugar that was directly used for the next reaction without further purification (60 mg, 0.27 mmol, quant. yield).

^1^H NMR (400 MHz, CD_3_OD) δ 4.61 (s, 1H, H1), 4.38 (d, *J* = 8.7 Hz, 1H, H2’), 3.98–3.90 (m, 1H, H2), 3.79 (dd, *J* = 12.0, 6.8 Hz, 1H, H6a), 3.74–3.63 (m, 2H, H3, H4), 3.60–3.52 (m, 1H, H5), 3.43–3.33 (m, 1H, H6b), 3.13 (dd, *J* = 13.0, 3.0 Hz, 1H, H3’a), 2.92 (dd, *J* = 13.1, 8.8 Hz, 1H, H3’b), 2.19 (ddd, *J* = 13.5, 6.3, 2.6 Hz, 1H, H1’a), 1.86–1.75 (m, 1H, H1’b).

^13^C NMR (101 MHz, D_2_O) δ 85.34 (CH), 80.54 (CH), 76.40 (CH), 76.09 (CH), 75.35 (CH), 70.23 (CH), 62.68 (CH_2_), 45.17 (CH_2_), 36.37 (CH_2_).

C_9_H_17_NO_5_; calcd. mass 219.24; ESI-MS: *m*/*z* 220.2 [M + H]^+^.

(2S,3aR,5R,6S,7S,7aR)-2-(aminomethyl)-5-(hydroxymethyl)hexahydro-2H-furo[3,2-b]pyran-6,7-diol **29b**

Compound **29b** was obtained following the same procedure reported for **28b**. Catalytic hydrogenolysis (41 mg, 0.123 mmol) afforded 27 mg (0.123 mmol, quant. yield) of amine **29b** that was used directly for the next reaction.

^1^H NMR (400 MHz, CD_3_OD) δ 4.46 (s, 1H, H1), 4.30 (dd, *J* = 8.7, 4.6 Hz, 1H, H2’), 4.01 (dd, *J* = 12.1, 8.3 Hz, 1H, H6a), 3.89–3.82 (m, 1H, H4), 3.75 (dt, *J* = 8.1, 3.6 Hz, 2H, H2, H5), 3.62 (dd, *J* = 12.1, 3.2 Hz, 1H, H6b), 3.54 (t, *J* = 4.9 Hz, 1H, H3), 3.13 (dd, *J* = 13.2, 3.6 Hz, 1H, H3’a), 3.05 (dd, *J* = 13.2, 5.0 Hz, 1H, H3’b), 2.51–2.38 (m, 1H, H1’a), 1.96 (s, 1H, H1’b).

^13^C NMR (101 MHz, D_2_O) δ 85.24 (CH), 80.52 (CH), 77.03 (CH), 75.40 (CH), 74.99 (CH), 70.02 (CH), 62.32 (CH_2_), 45.98 (CH_2_), 36.56 (CH_2_).

C_9_H_17_NO_5_; calcd. mass 219.24; ESI-MS: *m*/*z* 220.2 [M + H]^+^.

N-(((2R,3aR,5R,6S,7S,7aR)-6,7-dihydroxy-5-(hydroxymethyl)hexahydro-2H-furo[3,2-b]pyran-2-yl)methyl)-5-(dimethylamino)naphthalene-1-sulfonamide **3**

Bicyclic amine **29a** (60 mg, 0.27 mmol) was dissolved in MeOH (5 mL); Et_3_N (0.57 mmol, 2.1 eq) and dansyl chloride (0.41 mmol, 1.5 eq) were added and the reaction was stirred at r.t. until TLC (EtOAc/MeOH 9:1 and EtOAc/MeOH/H_2_O/AcOH 6:4:1:1) indicated the end of the reaction. After 3 h, the crude reaction was subjected to flash chromatography on silica gel (eluent EtOAc/MeOH 9.5:0.5) which afforded 51 mg (0.11 mmol, 41% yield) of compound **3**.

^1^H NMR (400 MHz, CD_3_OD) δ 8.56 (d, *J* = 8.5 Hz, 1H, CH Ar), 8.36 (d, *J* = 8.7 Hz, 1H, CH Ar), 8.19 (dd, *J* = 7.3, 1.1 Hz, 1H, CH Ar), 7.64–7.53 (m, 2H, CH Ar), 7.28 (d, *J* = 7.6 Hz, 1H, CH Ar), 4.40 (t, *J* = 7.6 Hz, 1H, H1), 4.11 (s, 1H, H2’), 3.75–3.60 (m, 3H, H6a, H2, H6b), 3.50 (dd, *J* = 9.5, 6.5 Hz, 1H, H3), 3.46 (dd, *J* = 10.7, 4.0 Hz, 1H, H5), 3.28–3.20 (m, 1H, H4), 3.00 (dd, *J* = 13.7, 4.6 Hz, 1H, H3’a), 2.93 (dd, *J* = 13.7, 5.3 Hz, 1H, H3’b), 2.88 (s, 6H, -N(CH_3_)_2_), 1.91 (ddd, *J* = 13.2, 6.5, 3.6 Hz, 1H, H1’a), 1.77–1.63 (m, 1H, H1’b).

^13^C NMR (101 MHz, CD_3_OD) δ 153.15 (Cq Ar), 137.14 (Cq Ar), 131.16 (Cq Ar), 131.12 (C Ar), 130.90 (Cq Ar), 130.01 (C Ar), 129.10 (C Ar), 124.30 (C Ar), 120.54 (C Ar), 116.41 (C Ar), 84.35 (C2’), 79.21 (C1), 77.47 (C2), 74.94 (C4), 74.31 (C3), 69.52 (C5), 62.29 (C6), 47.46 (C3’), 45.80 ((CH_3_)_2_N-), 35.01 (C1’).

C_21_H_28_N_2_O_7_S; calcd. mass 452.52; ESI-MS: *m*/*z* 453.4 [M + H]^+^. [α]_D_^20^ +12 (c = 0.3, MeOH).

N-(((2S,3aR,5R,6S,7S,7aR)-6,7-dihydroxy-5-(hydroxymethyl)hexahydro-2H-furo[3,2-b]pyran-2-yl)methyl)-5-(dimethylamino)-naphthalene-1-sulfonamide **4**

Compound **29b** (27 mg, 0.123 mmol) was converted into dansyl-sulphonamide derivative **4** (32 mg, 0.07 mmol, 57% yield) using the same procedure described for the diastereoisomer **29a**.

^1^H NMR (400 MHz, CD_3_OD) δ 8.56 (d, *J* = 8.5 Hz, 1H, CH Ar), 8.36 (d, *J* = 8.7 Hz, 1H, CH Ar), 8.20 (dd, *J* = 7.3, 1.1 Hz, 1H, CH Ar), 7.59 (td, *J* = 8.8, 7.6 Hz, 2H, CH Ar), 7.28 (d, *J* = 7.2 Hz, 1H, CH Ar), 4.44 (dd, *J* = 12.0, 5.3 Hz, 1H, H1), 3.94–3.83 (m, 1H, H2’), 3.72 (dd, *J* = 12.0, 6.4 Hz, 1H, H6a), 3.64 (dd, *J* = 12.4, 3.2 Hz, 1H, H6b), 3.62–3.53 (m, 2H, H2, H3), 3.48–3.41 (m, 1H, H5), 3.36–3.27 (m, 1H, H4), 3.02 (dd, *J* = 5.4, 2.4 Hz, 2H, H3’a, b), 2.88 (s, 6H, -N(CH_3_)_2_), 2.00 (dt, *J* = 13.7, 7.0 Hz, 1H, H1’a), 1.71 (ddd, *J* = 13.1, 7.5, 5.3 Hz, 1H, H1’b).

^13^C NMR (101 MHz, CD_3_OD) δ 153.14 (Cq Ar), 136.95 (Cq Ar), 131.16 (Cq Ar), 131.14 (C Ar), 130.87 (Cq Ar), 130.06 (C Ar), 129.20 (C Ar), 124.28 (C Ar), 120.49 (C Ar), 116.43 (C Ar), 83.95 (C2’), 78.88 (C1), 78.23 (C2), 75.48 (C4), 74.92 (C3), 69.49 (C5), 62.30 (C6), 47.99 (C3’), 45.79 ((CH_3_)_2_N-), 34.88 (C1’).

C_21_H_28_N_2_O_7_S; calcd. mass 452.52; ESI-MS: *m*/*z* 453.4 [M + H]^+^. [α]_D_^20^ +60 (c = 0.3, MeOH).

## 4. Conclusions

In this work, we have described the design and synthesis of glycoderivatives, which are potential SGLT1 ligands that could act as cytoprotective agents and serve as molecular/chemical tools for studying the SGLT1-mediated cytoprotection process. The mechanism underlying this phenomenon is still unclear, particularly how *C*-glucoside **1**, when interacting with SGLT1, acts as an anti-inflammatory/protective agent. The synthesis of the library involved introducing modifications to the sugar region and the spacer between the sugar and the aromatic group, thus expanding the diversity of chemical structures. This builds upon the first library of glycoderivatives targeting SGLT1, which was previously reported. The results obtained suggest that the aromatic/dansyl residue plays a primary role in the final cytoprotective effect, while the role of the sugar portion remains unclear.

## Data Availability

Data are contained within this article or in Supplementary Materials.

## Supplementary Materials

The following supporting information can be downloaded at https://www.mdpi.com/article/10.3390/molecules29215067/s1, ^1^H-NMR, 2D NOESY and ^13^C-NMR spectra of synthesized compounds.
