# Supplementary material for: Synthesis of a Small Library of Glycoderivative Putative Ligands of SGLT1 and Preliminary Biological Evaluation
Source: Molecules. 2024 Oct 26;29(21):5067. doi: 10.3390/molecules29215067 (PMC11547630; doi:10.3390/molecules29215067)
Supplement: Supplementary file 1 [file molecules-29-05067-s001.zip › Molecules-3240244-Supplementary materials.pdf]

molecules

Synthesis of a Small Library of Glycoderivative Putative Ligands of SGLT1 and Preliminary Biological Evaluation

Giuseppe D'Orazio[1] and Barbara La Ferla[2,*]

Supplementary Materials

^{1}H-NMR, 2D NOESY and ^{13}C-NMR spectra of synthesized compounds.

- Compound 13
- Compound 14
- Compound 15
- Compound 16
- Compound 17
- Compound 18
- Compound 2
- Compound 6
- Compound 22
- Compound 23
- Compound 4
- Compound 28a
- Compound 28b
- Compound 29a
- Compound 29b
- Compound 3
- Compound 4

Compound 13

^{1}H-NMR (400 MHz, $CDCl_3$)

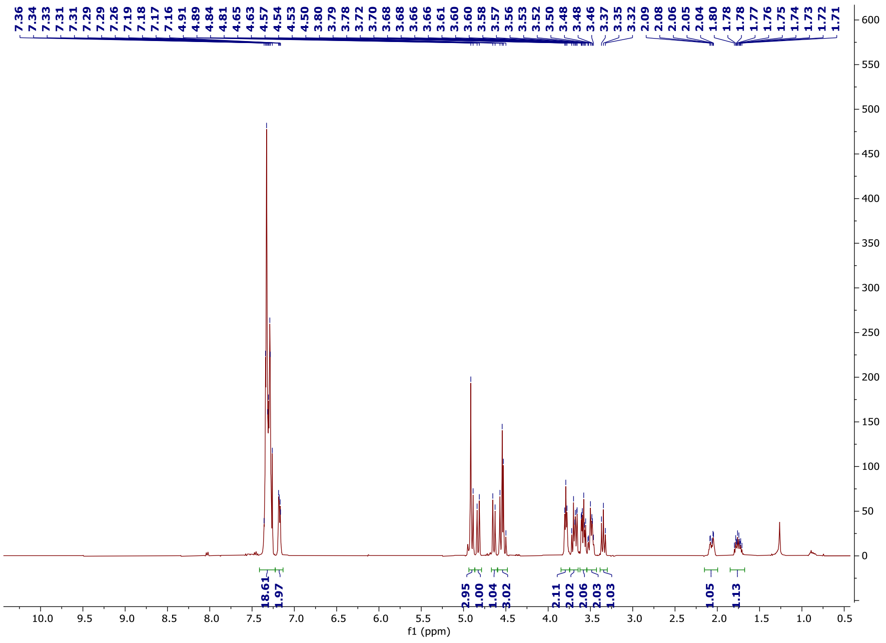

^{13}C-NMR (101 MHz, $CDCl_3$)

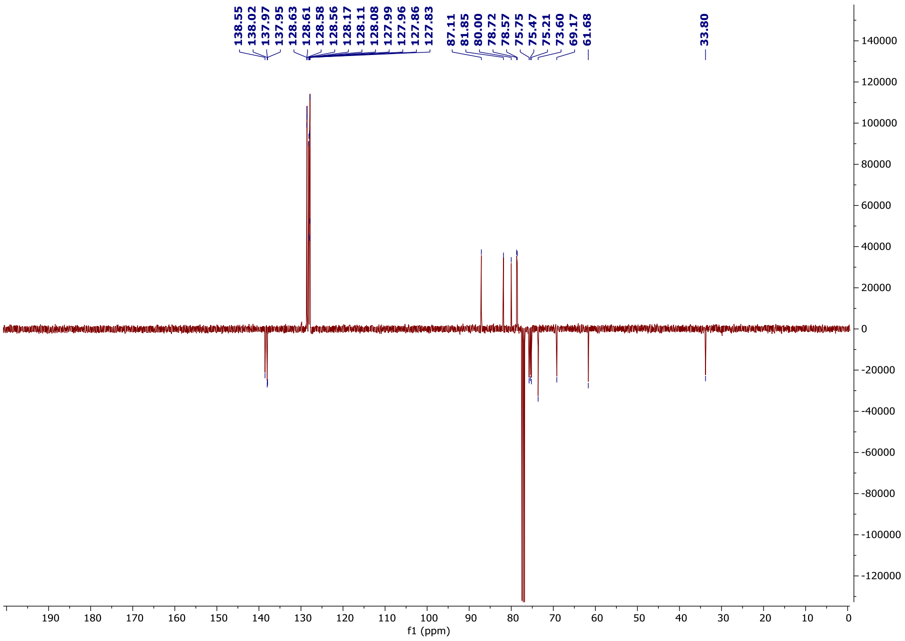

Compound 14

^{1}H-NMR (400 MHz, $CDCl_3$)

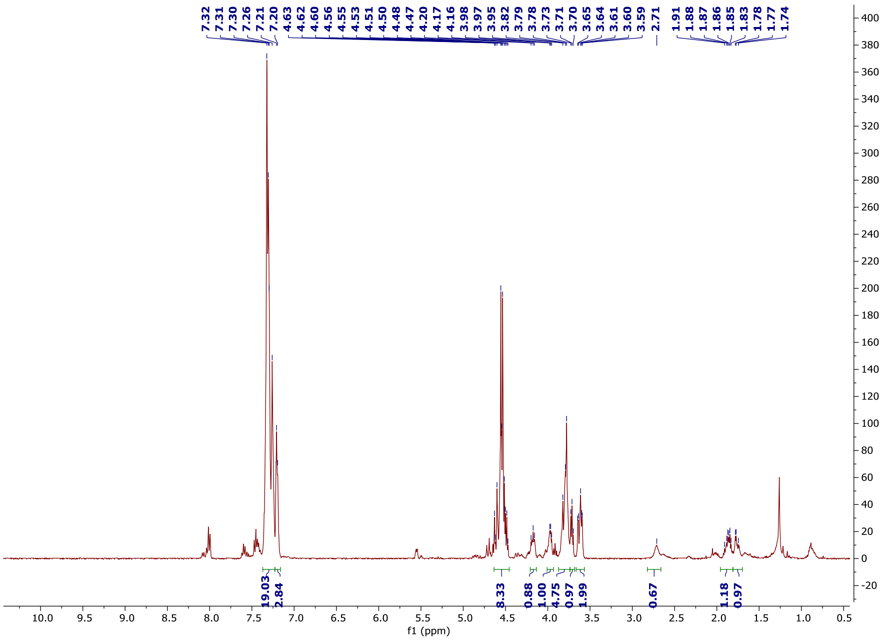

^{13}C-NMR (101 MHz, $CDCl_3$)

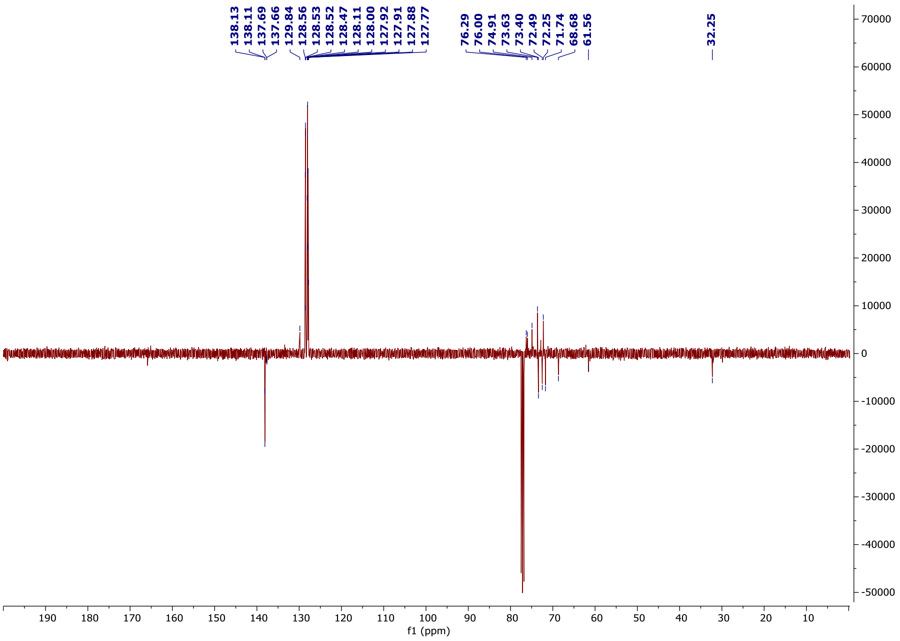

Compound 15

^{1}H-NMR (400 MHz, CDCl$_3$)

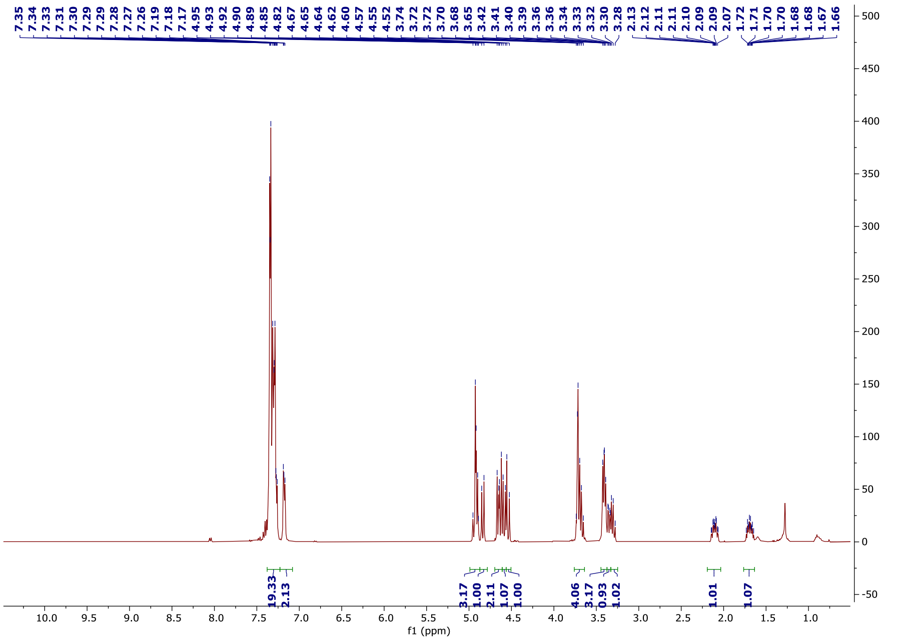

^{13}C-NMR (101 MHz, CDCl$_3$)

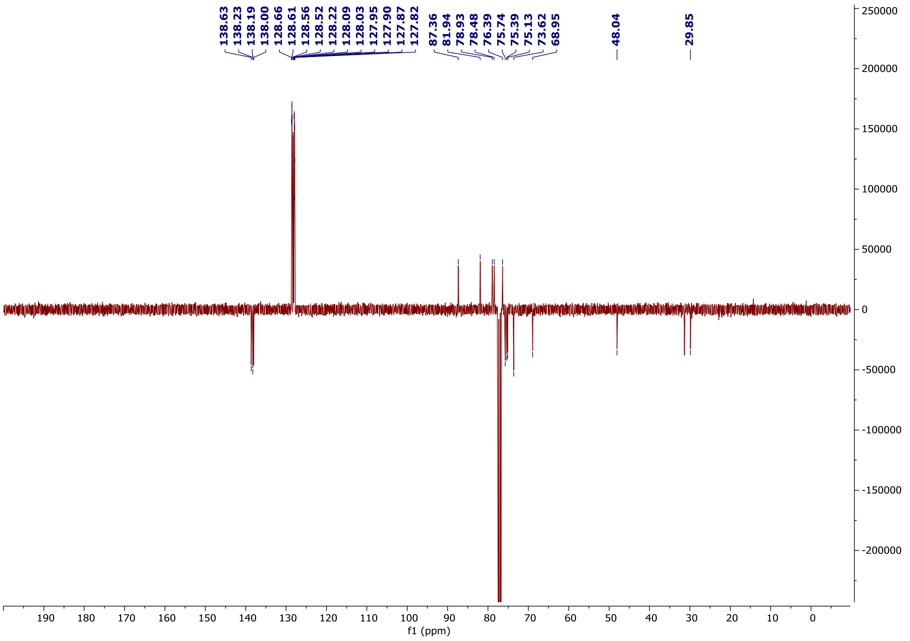

Compound 16

^{1}H-NMR (400 MHz, $CDCl_3$)

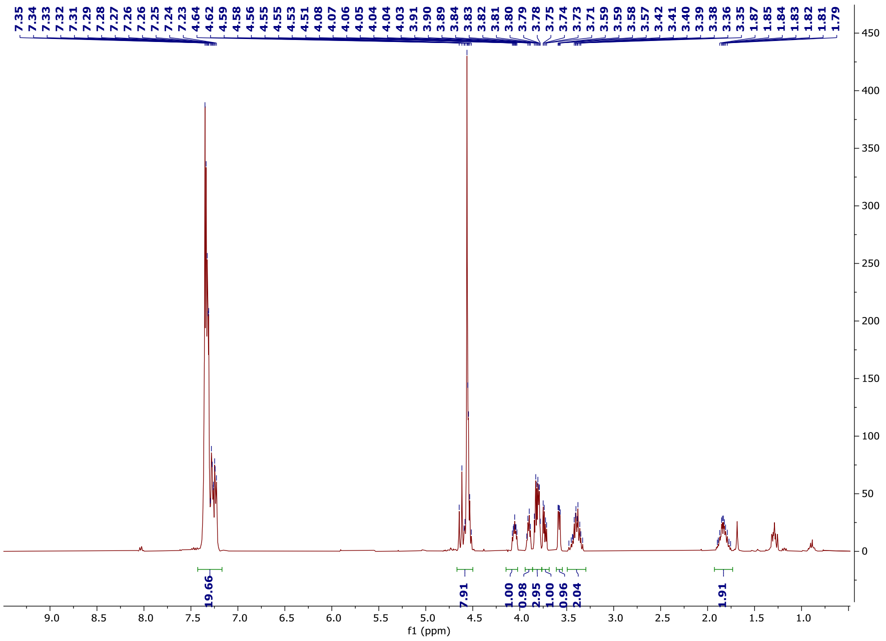

^{13}C-NMR (101 MHz, $CDCl_3$)

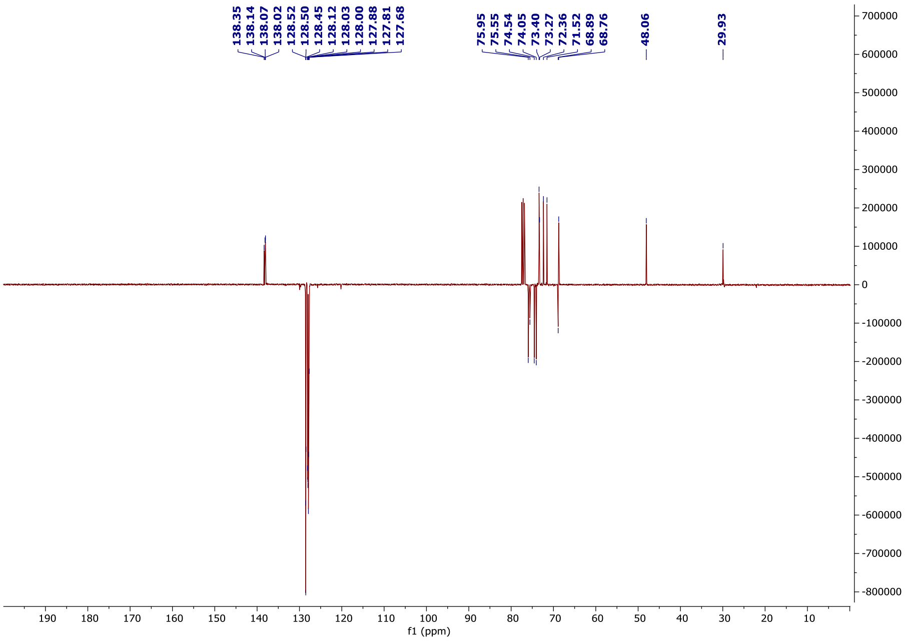

Compound 17

^{1}H-NMR on crude material (400 MHz, D_2O)

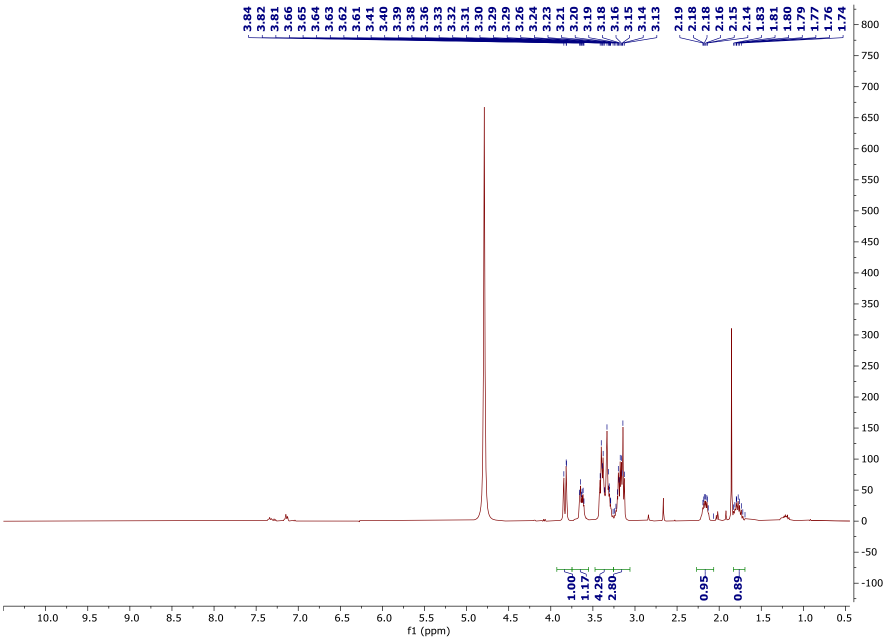

Compound 18

^{1}H-NMR on crude material (400 MHz, D_2O)

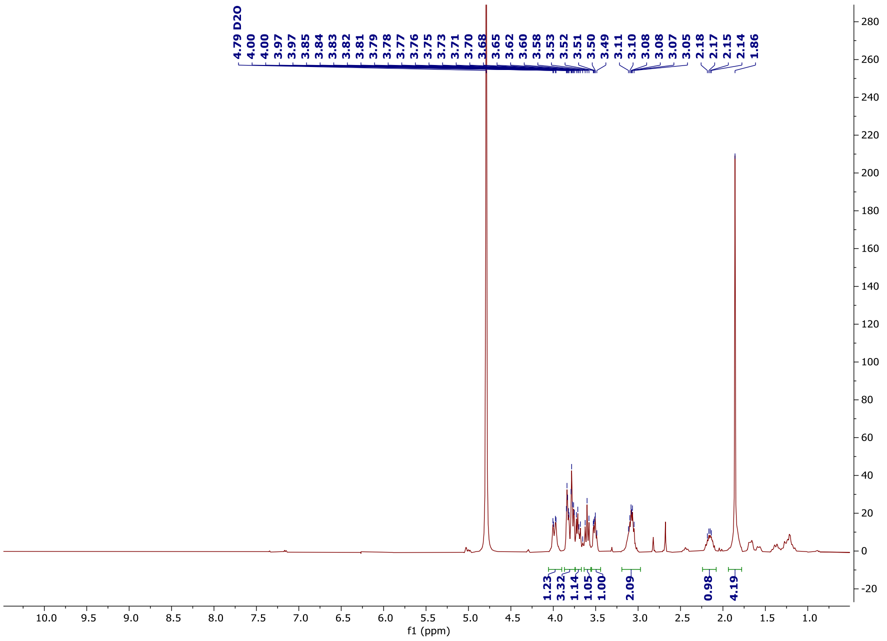

Compound 2

^{1}H-NMR (400 MHz, CD_3OD)

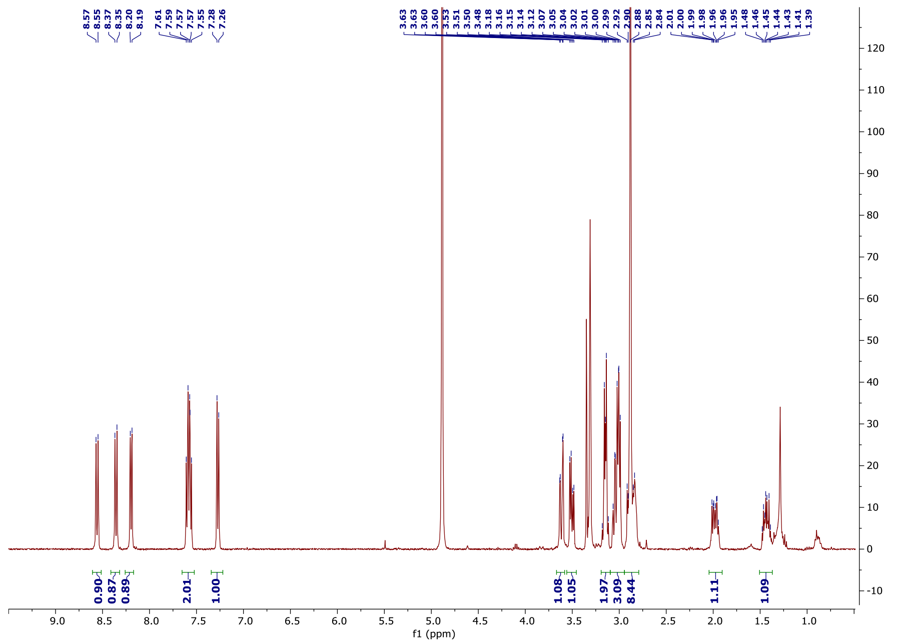

^{13}C-NMR (101 MHz, CD_3OD)

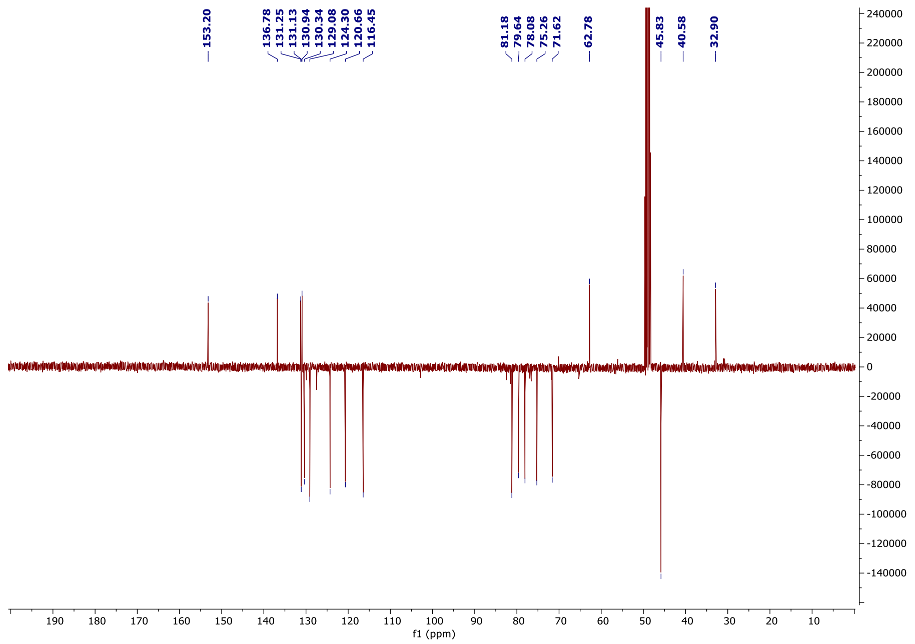

Compound 6

^{1}H-NMR (400 MHz, CD_3OD)

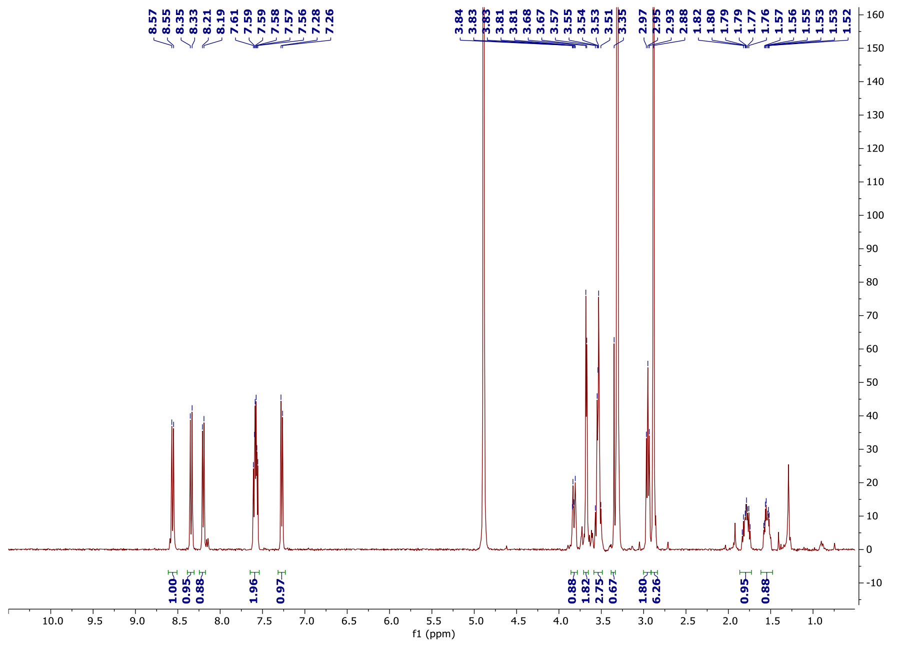

^{13}C-NMR (101 MHz, D_2O)

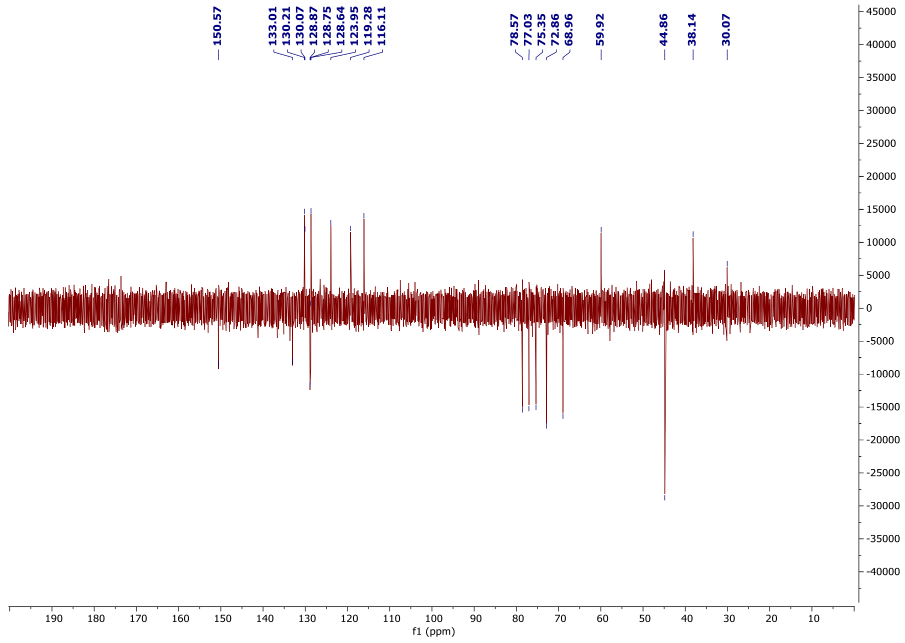

Compound 22

^{1}H-NMR on crude material (400 MHz, $CDCl_3$)

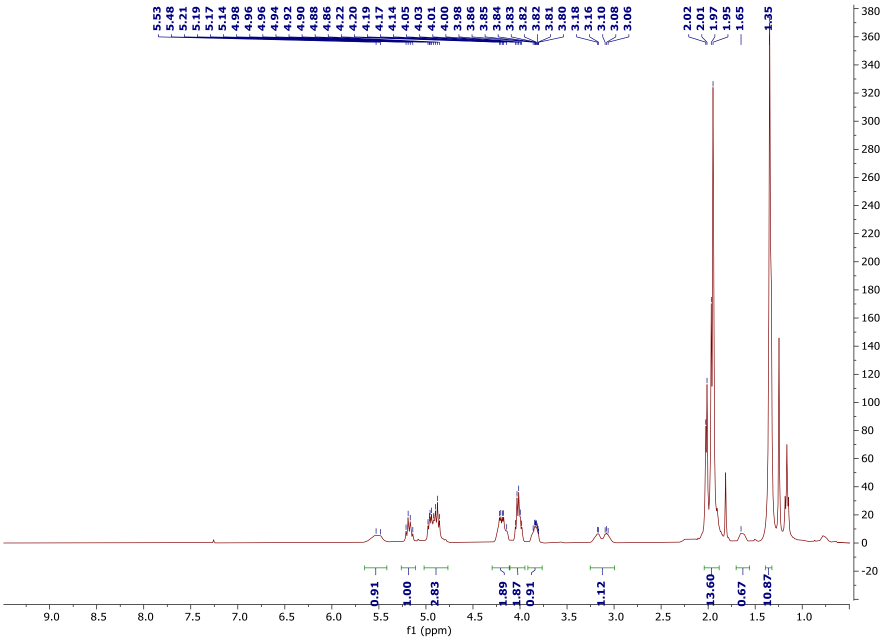

^{13}C-NMR on crude material (101 MHz, $CDCl_3$)

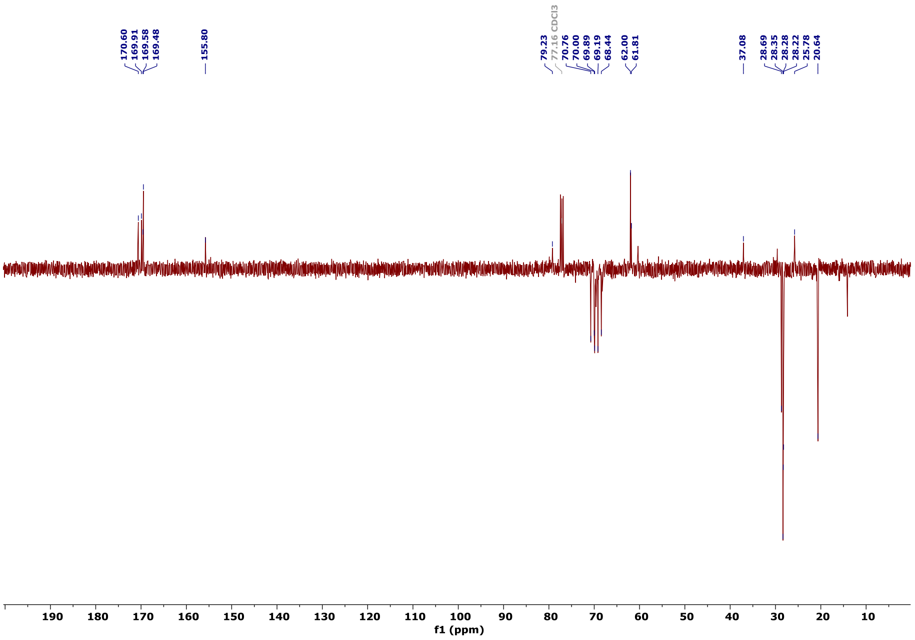

Compound 23

^{1}H-NMR on crude material (400 MHz, $CDCl_3$)

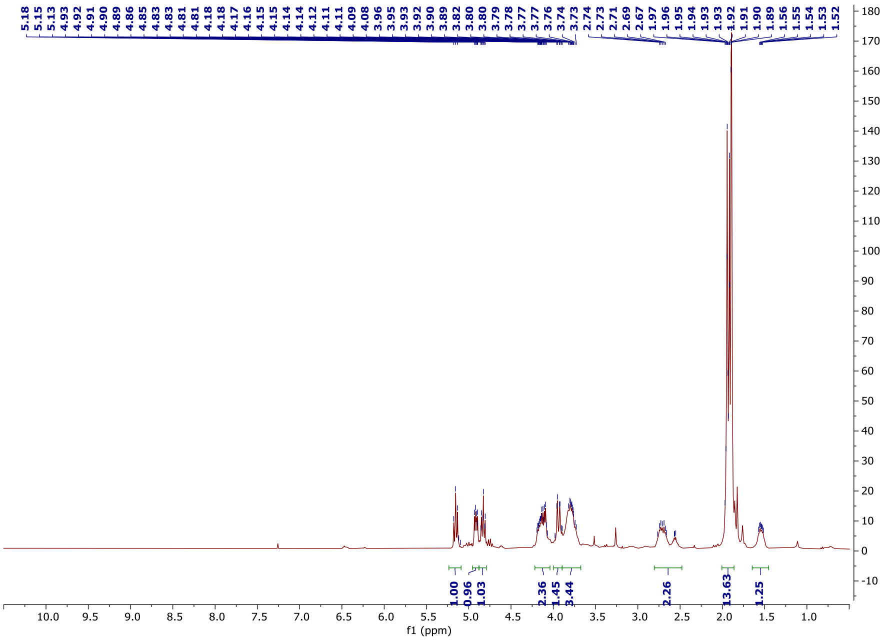

^{13}C-NMR on crude material (101 MHz, $CDCl_3$)

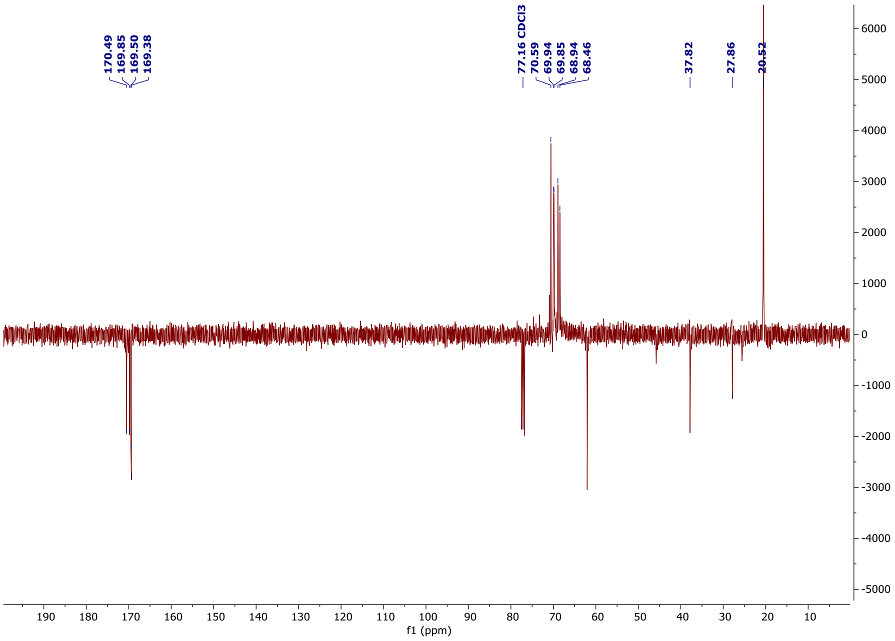

Compound 4

^{1}H-NMR (400 MHz, CD_3OD)

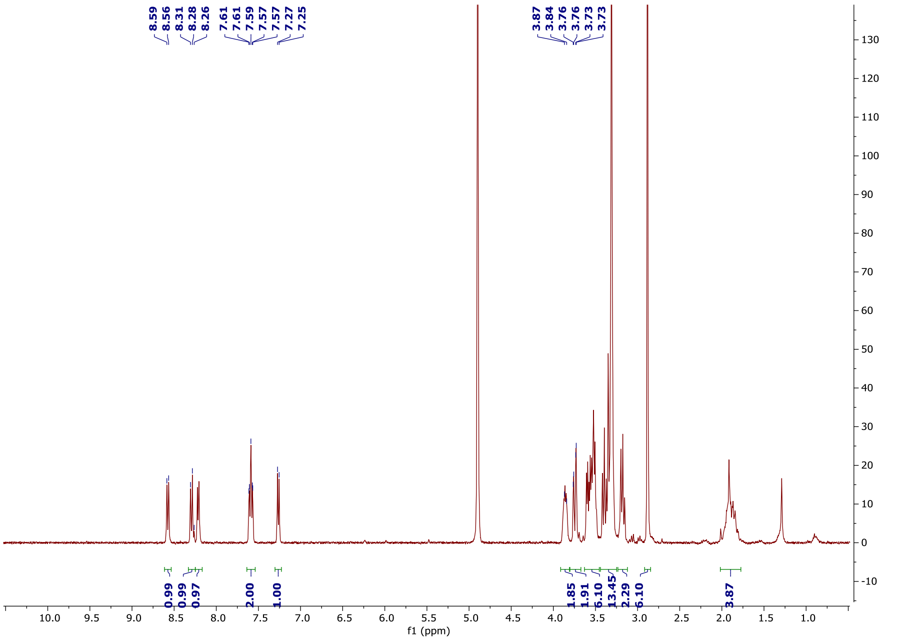

^{13}C-NMR (101 MHz, CD_3OD)

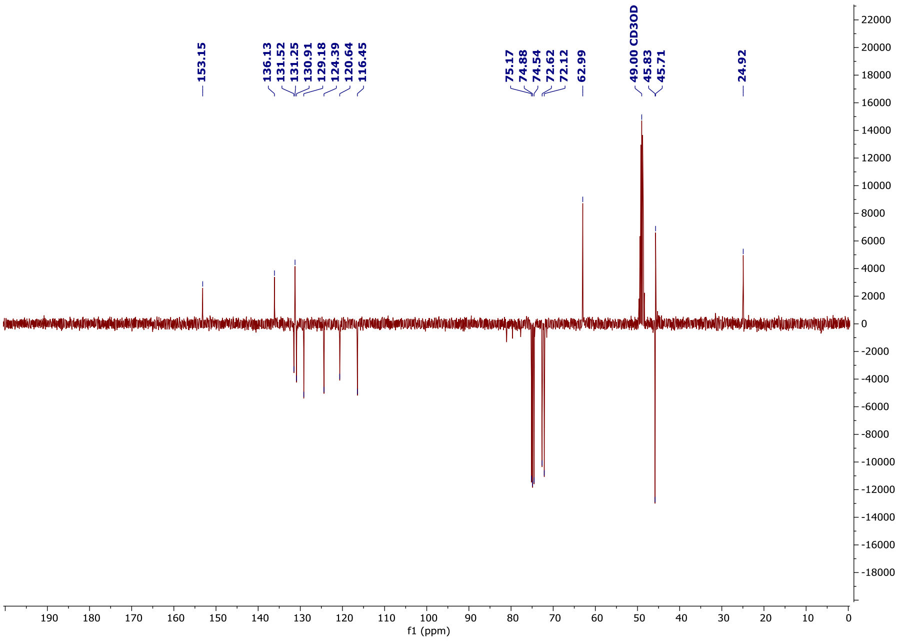

Compound 28a

^{1}H-NMR (400 MHz, CDCl$_3$)

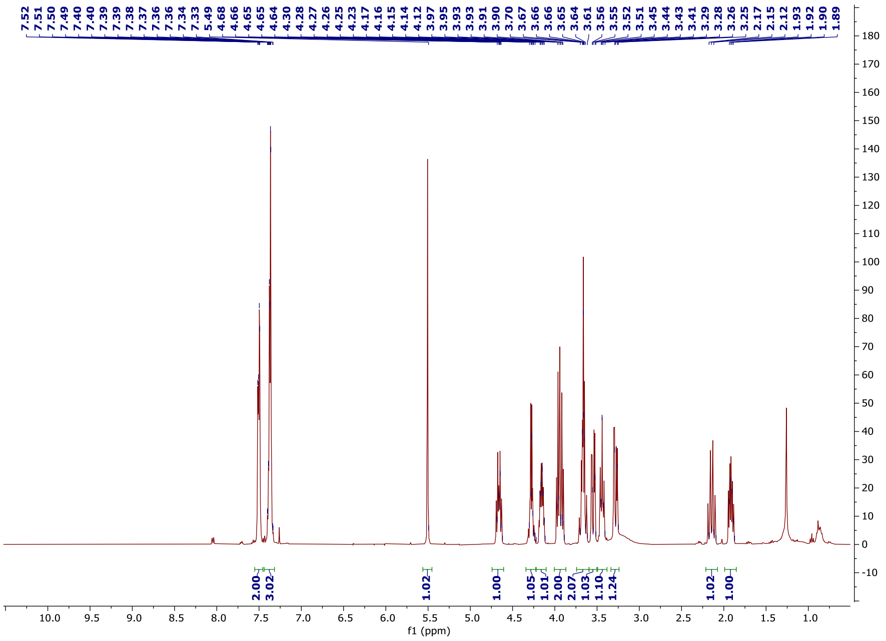

2D ^{1}H NOESY (400 MHz, CDCl$_3$)

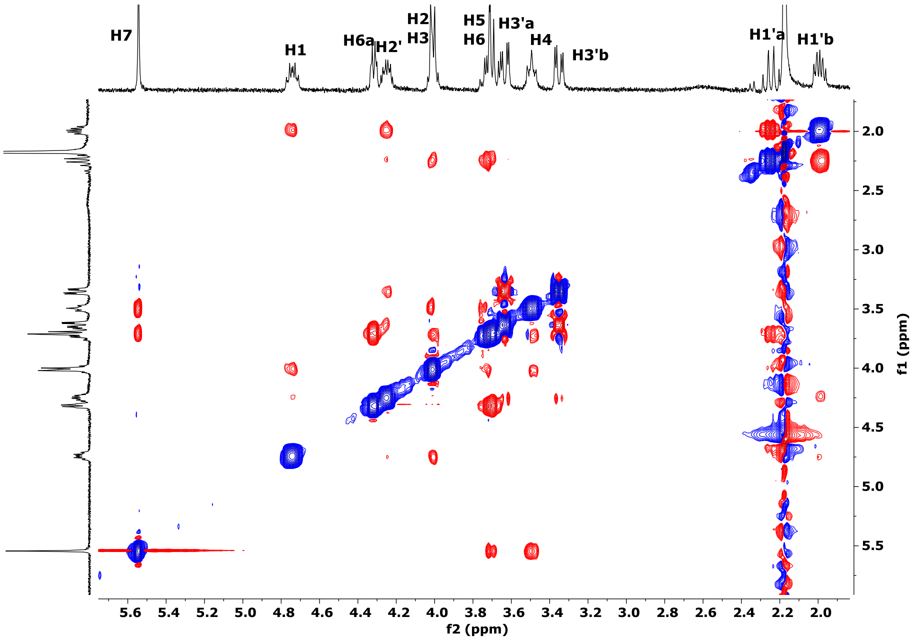

^{13}C-NMR (101 MHz, $CDCl_3$)

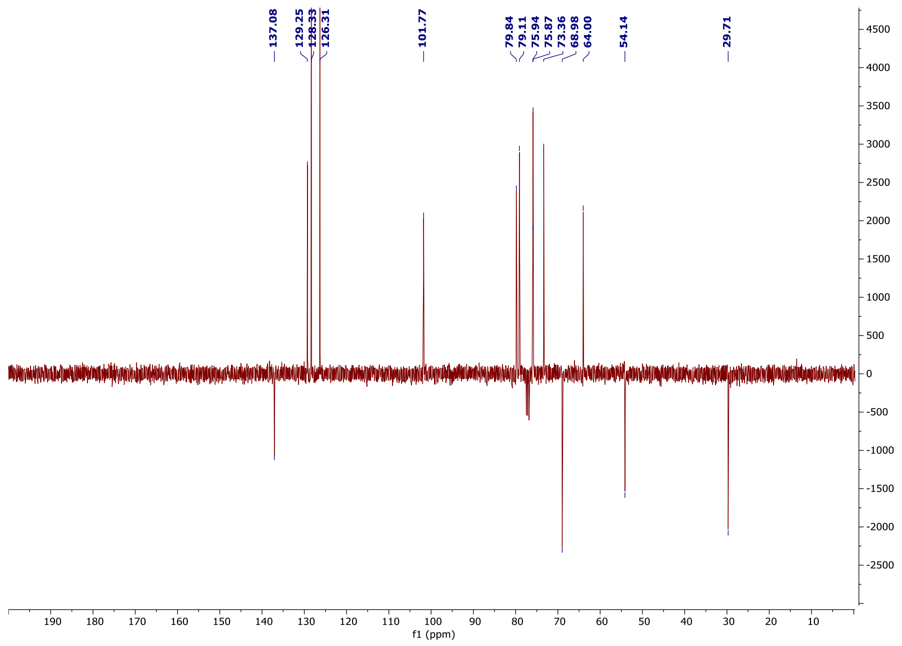

Compound 28b

^{1}H-NMR (400 MHz, $CDCl_3$)

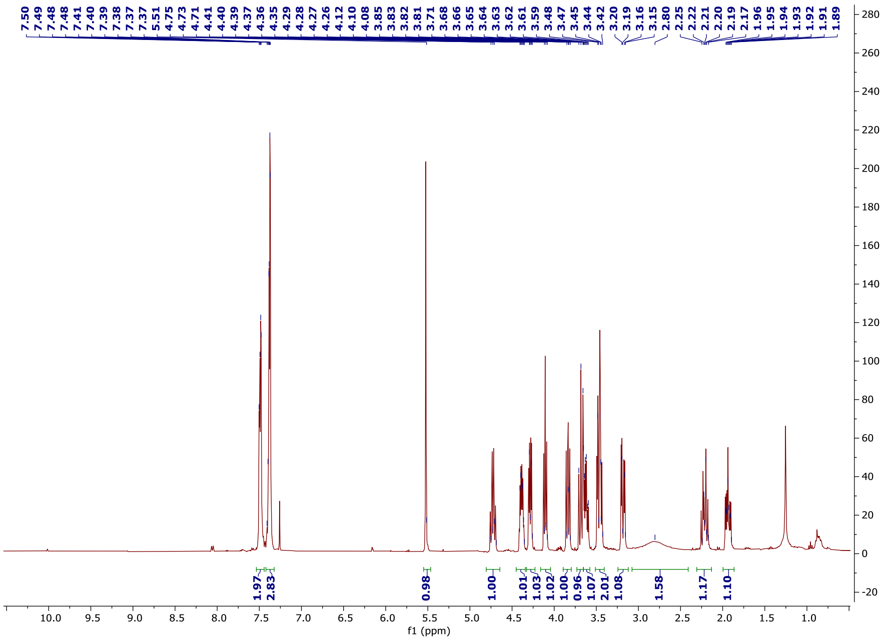

2D ^{1}H NOESY (400 MHz, $CDCl_3$)

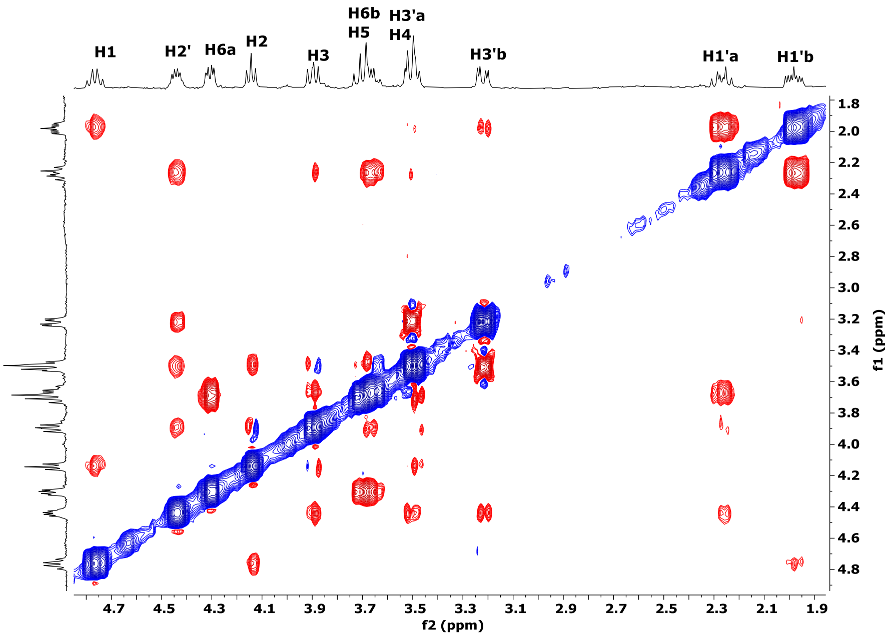

^{13}C-NMR (101 MHz, $CDCl_3$)

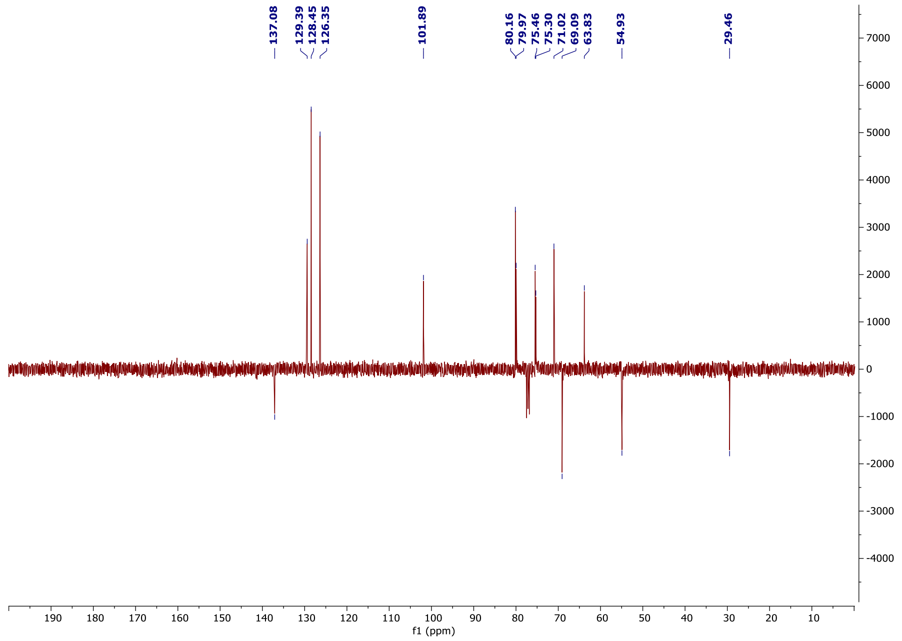

Compound 29a

^{1}H-NMR (400 MHz, $CDCl_3$)

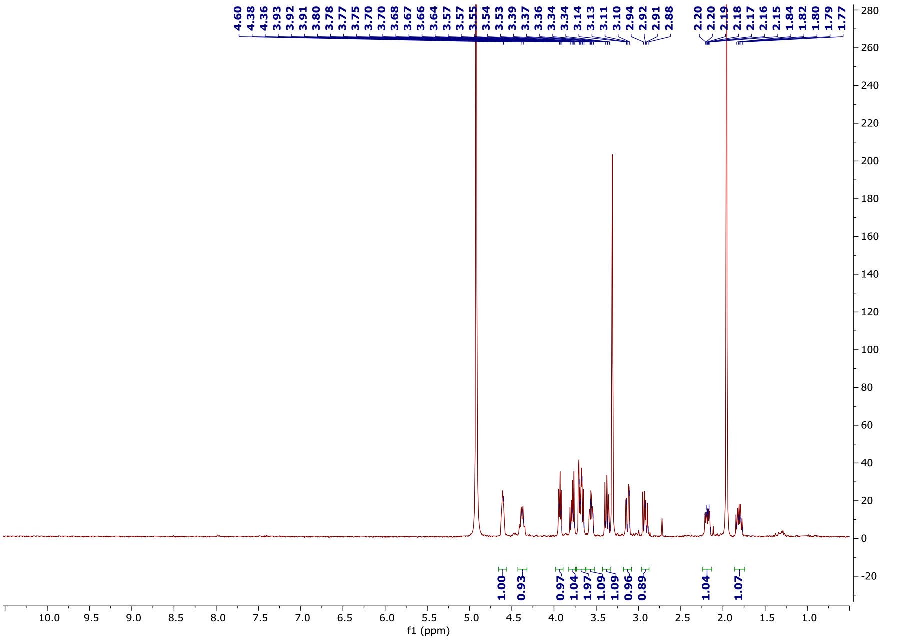

^{13}C-NMR (101 MHz, $CDCl_3$)

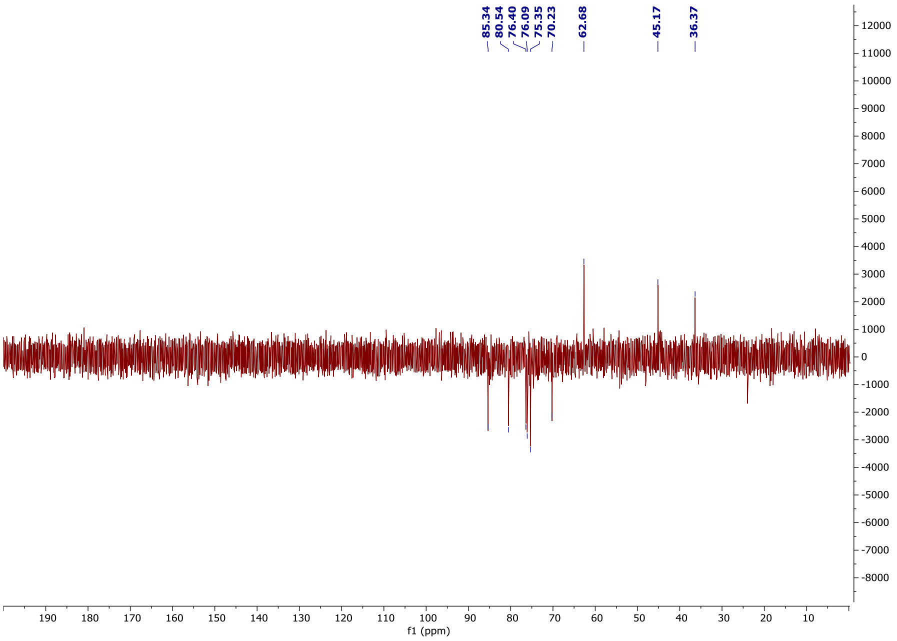

Compound 29b

^{1}H-NMR (400 MHz, $CDCl_3$)

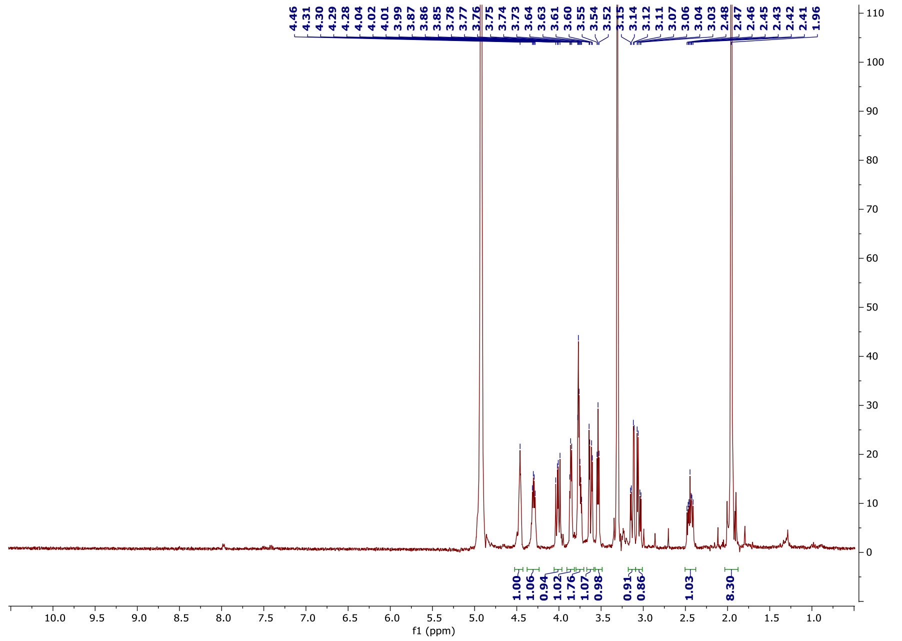

^{13}C-NMR (101 MHz, $CDCl_3$)

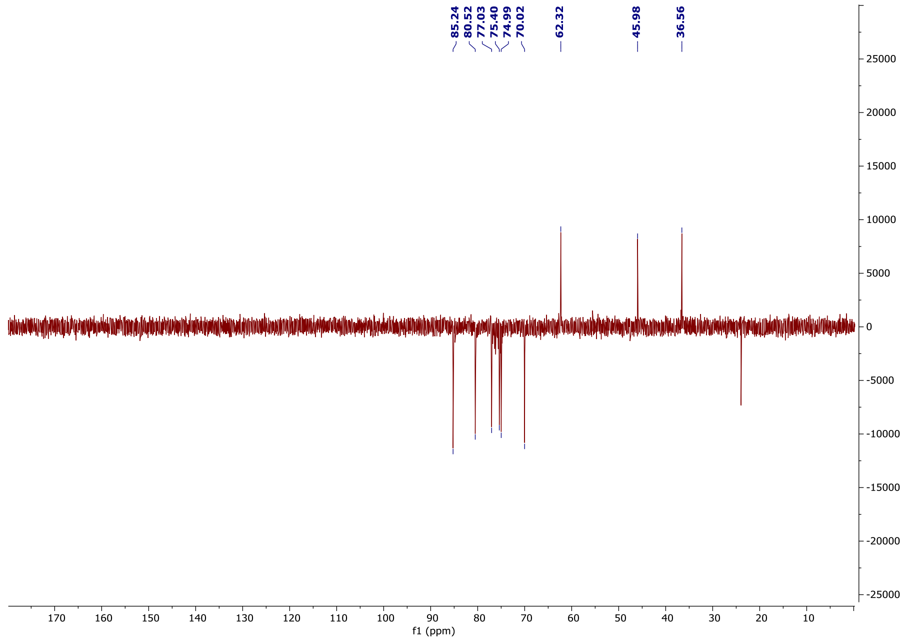

Compound 3

^{1}H-NMR (400 MHz, CD_3OD)

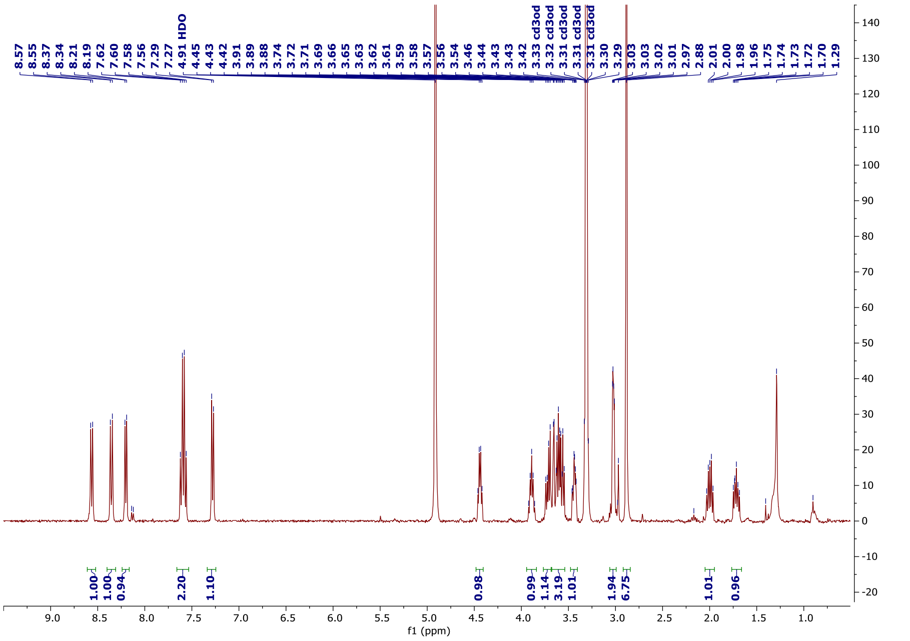

^{13}C-NMR (101 MHz, CD_3OD)

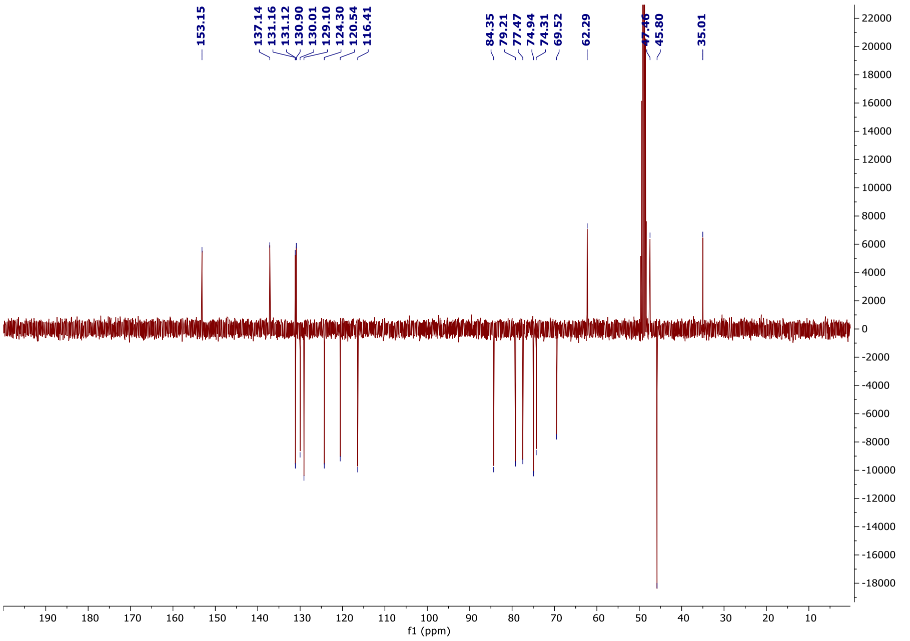

Compound 4

^{1}H-NMR (400 MHz, CD_3OD)

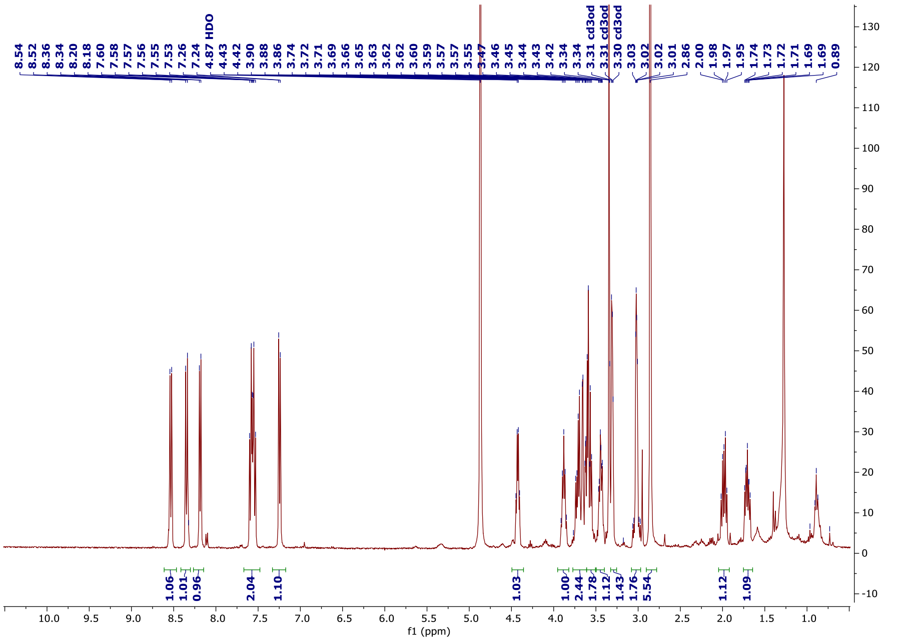

^{13}C-NMR (101 MHz, CD_3OD)

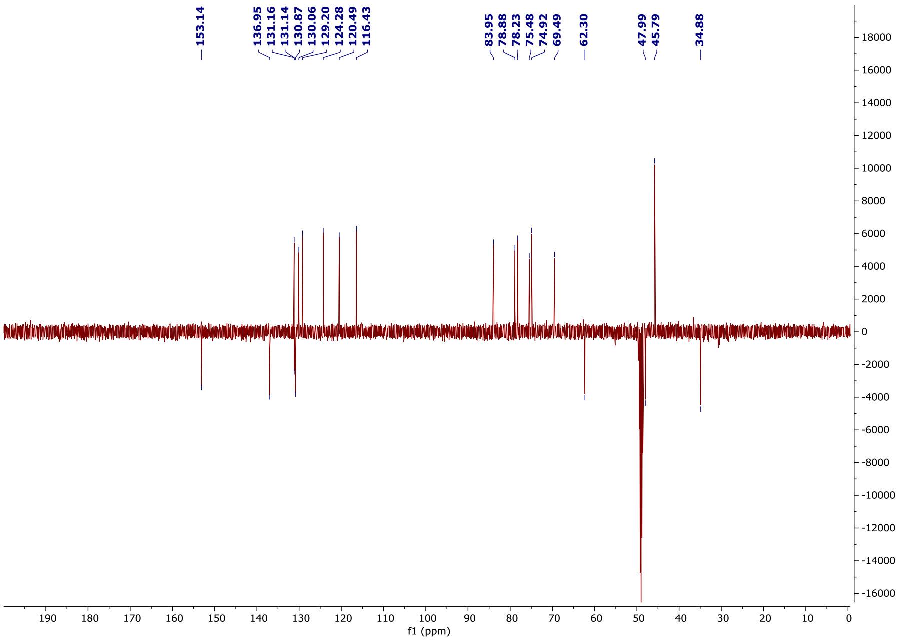

Disclaimer/Publisher's Note: The statements, opinions and data contained in all publications are solely those of the individual author(s) and contributor(s) and not of MDPI and/or the editor(s). MDPI and/or the editor(s) disclaim responsibility for any injury to people or property resulting from any ideas, methods, instructions or products referred to in the content.
